# G-quadruplexes in MTOR and induction of autophagy

**DOI:** 10.1038/s41598-024-52561-y

**Published:** 2024-01-30

**Authors:** Piyali Majumder, Chinmayee Shukla, Arjun Arya, Shubham Sharma, Bhaskar Datta

**Affiliations:** 1 Department of Biological Sciences and Engineering, Indian Institute of Technology Gandhinagar, Palaj, Gandhinagar, Gujarat 382355 India; 2 Department of Chemistry, Indian Institute of Technology Gandhinagar, Palaj, Gandhinagar, Gujarat 382355 India

**Keywords:** Biochemistry, Chemical biology

## Abstract

G-quadruplex (G4) structures have emerged as singular therapeutic targets for cancer and neurodegeneration. Autophagy, a crucial homeostatic mechanism of the cell, is often dysregulated in neurodegenerative diseases and cancers. We used QGRS mapper to identify 470 G4 sequences in MTOR, a key negative regulator of autophagy. We sought to identify a functional context by leveraging the effect of G4-targeting ligands on MTOR G4 sequences. The effect of Bis-4,3, a G4 selective dimeric carbocyanine dye, was compared with the known G4-stabilizing activity of the porphyrin, TMPyP4 in HeLa and SHSY-5Y cells. Our results show that treatment with G4-selective ligands downregulates MTOR RNA and mTOR protein expression levels. This is the first report describing G4 motifs in MTOR. This study indicates a possible role of G4 stabilizing ligands in induction of autophagy by downregulation of mTOR levels, albeit not precluding MTOR independent pathways.

## Introduction

The perceptions surrounding G-quadruplexes (G4) have undergone a significant makeover over the past two decades. While initially viewed as unusual structures that could be produced in in vitro confinement, the past two decades have provided mounting evidence on their physiological relevance. Computational analysis has predicted over 350,000 putative quadruplex forming sequences while G4-seq identified over 700,000 G4 structures in the human genome^1,2^. Thermodynamic and kinetic studies of DNA G-quadruplexes reveal higher stability and slower rates of unfolding in comparison to double-stranded DNA^3^. In vivo studies using G4-selective antibodies have validated the formation of G-quadruplex structures in cells^4^. G4s have been experimentally mapped in human genomic DNA and are found to be enriched at promoter sites, 5′ and 3′-UTRs and first introns suggesting deep involvement in gene regulatory events^5,6^. As a result, G4s have emerged as an attractive therapeutic target for several disease conditions and disorders^7^.

G4 structures have been implicated in modulating the transcription and translation of several genes such as c-MYC, NRAS, KRAS, BCL-2 and c-KIT^8–12^. G4-stabilization in oncogene promoter regions has been established as an effective mechanism for the down-regulation of genes involved in tumour survival and metastasis^13^. In particular, small molecule-mediated stabilization of such G4 elements elicits transcriptional control that ultimately halts tumour progression. Targeting G4s appears to be a promising anti-cancer approach because of the over-expression of putative quadruplex-containing genes in specific cancers^14^. Generally, sequence information of guanine-rich regions and in silico identification of putative quadruplex forming sequences are used to study in vitro and in cellulo effects of G4 targeting ligands. The small molecule-mediated stabilization of G4s is reported to affect global processes such as apoptosis, autophagy, senescence and signalling pathways such as the hypoxia-inducible factor pathway^15,16^. Our interest in DNA G4 motifs originated from the presence of these structures in genes having prominent regulatory and functional roles in cellular homeostasis. In this regard, mTOR (mammalian target of rapamycin) functions as a crucial regulator of cellular homeostasis by acting as an antagonistic regulator of autophagy^17–19^. Autophagy is characterized by sequestration and digestion of cellular components in a double membrane vesicle to maintain a homeostatic balance^20^. The stress response generated by autophagy could help tumour cell growth or serve as a tumour suppressor by preventing the accumulation of damaged proteins and organelles^21^. Several autophagy inducers have been used as therapeutics for cancer, showing chemosensitivity in apoptosis incompetent cancer cells^22^. We were interested in investigating the presence of G4 structures in the *MTOR* gene. In this work, in silico identification of putative quadruplex forming sequences in the *MTOR* gene is followed by suitable in vitro characterization. Further, we have used G4-targeting small molecules to assess the potential role of G4-motifs of *MTOR* in gene expression modulation, thereby providing a new perspective on autophagy induction.

## Results and discussion

### The abundance of G-quadruplex structures in the *MTOR* gene

**Figure 1:**
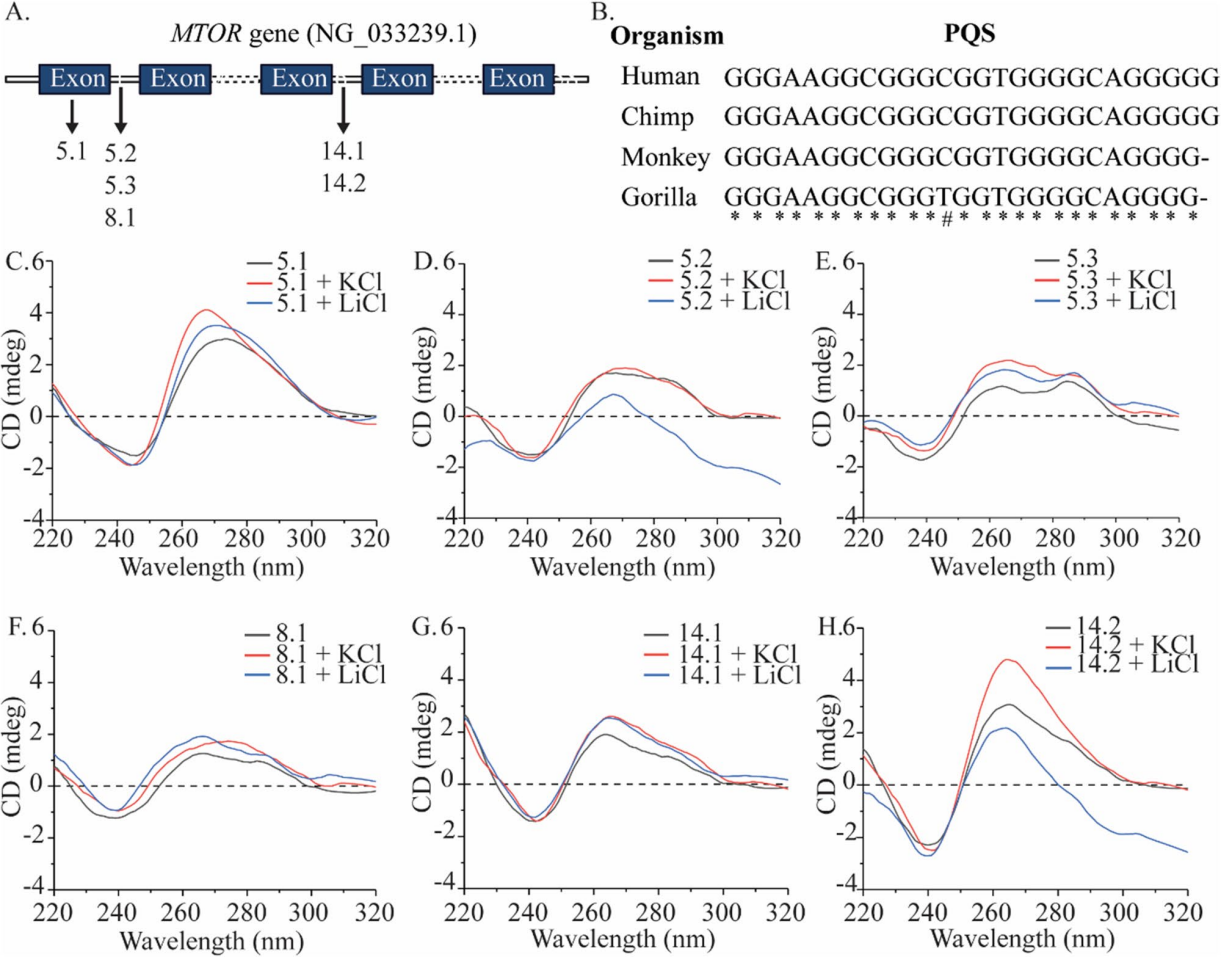
(**A**) The localization of various G-rich regions of the *MTOR* gene on chromosome 1. (**B**) Multiple sequence alignment of the 5.1 putative quadruplex forming sequence in MTOR homologs of human, chimp, monkey and gorilla. CD spectra in absence of ions and presence of monovalent cations K^+^ and Li^+^ (100 mM) of (**C**) Oligo 5.1, (**D**) Oligo 5.2, (**E**) Oligo 5.3, (**F**) Oligo 8.1, (**G**) Oligo 14.1, (**H**) Oligo 14.2.

mTOR is a critical regulator of various metabolic pathways inside the cell^17^. We used the QGRS mapper tool to assess putative G-quadruplex (G4) forming sites^23^. This bioinformatics analysis was performed using the entire sequence from the RefSeqGene (LRG_734) for the *MTOR* gene (NG_033239.1) on chromosome 1. QGRS mapper analysis identified 470 putative quadruplex forming sequences spread across the *MTOR* gene. Upstream elements have traditionally been implicated in regulating the replication or transcription of genes^24,25^. In the *MTOR* gene, three G-rich sites emerge from the QGRS bioinformatics analysis. These three G-rich sites are at 5 kb, 8 kb and 14 kb from nucleotide 1 (Fig. 1A). When transcribed, G-rich site 5.1, present in the 5′ UTR of the pre-mRNA, resides in the first exon, and can potentially regulate the translation of the *MTOR* RNA^26^. We performed multiple sequence alignment on MTOR homologs in human, monkey, chimp and gorilla and found that the exonic G4 is conserved across the species and could be of significance^27^ (Fig. 1B). Evolutionary conserved quadruplex motifs are strongly correlated for their functional relevance in gene regulation and grant us a potential intervention point^25,28^. G-rich sites 5.2, 5.3 and 8.1 reside in the first intron and could assist the loading of transcriptional regulators by leveraging their proximity to the core transcription complex^29^. Oligos 14.1 and 14.2 reside between exons 6 and 7 (NG_033239.1). G4 motifs in the DNA can play a subtle but substantive role in replication and gene expression modulation by influencing transcription and translation^30^.

We investigated the quadruplex-forming potential of the sequences identified by QGRS mapper analysis via Circular Dichroism (CD) spectroscopy on cognate oligonucleotides (Table S1). CD is capable of informing about the presence and topology of G4 structures^31^. CD spectroscopy was performed for six oligonucleotides across three G-rich sites in the presence of KCl (stabilizing effect) or LiCl (destabilizing/neutral effect) and in the absence of monovalent cations (Fig. 1C–H).

A broad CD spectra corresponding to a parallel G-quadruplex was observed in Oligo 5.1 in absence of monovalent ions and in presence of LiCl. The ~ 264 nm peak narrowed and increased in ellipticity in presence of K^+^ ions. Oligo 5.2 showed destabilization in presence of Li^+^ ions while a hybrid G4 was observed in absence of monovalent ions. Upon addition of K^+^ ions, the CD spectra shifted to a broad parallel G4, in a propeller form^32,33^. Peaks corresponding to hybrid G4s were observed for oligo 5.3 in presence of K^+^, Li^+^ or absence of ions, however with decreasing order of ellipticity. Hybrid G4s observed in oligo 8.1 in absence of ions and presence of Li^+^ shifted to a broad parallel G4 upon addition of K^+^ ions. Peaks corresponding to parallel G4s (propeller-type) were observed in presence of K^+^ and Li^+^ for oligo 14.1, indicating that Li^+^ ions played a neutral effect on the quadruplex structure. These peaks decreased in ellipticity in absence of monovalent ions. Oligo 14.2 showed peaks corresponding to a parallel G4 in presence of K^+^ ions. A decrease in ellipticity was observed in absence of ions while Li^+^ destabilized the structure. Thus, the CD spectra of the oligonucleotides reflect their inherent G4-forming propensity and their variable strength when no monovalent cations are present and in the presence of K^+^ or Li^+^ ions^34^.

We next studied oligos-bearing mutations that disrupt the pattern of G-tracts, which in turn is likely to influence association into G-quadruplexes. The sequences used for this part of the study are listed in Supplementary Table 1. CD spectra of the mutant oligos indicate a deviation from the topology adopted by the wild-type sequence in each case. In particular, the mutant versions of oligos 5.1, 5.3, 8.1 and 14.2 display dissimilar CDs from their wild-type counterparts and are consistent with a loss of the native G-quadruplex topology. Other than the 5.1R which displayed a much lower parallel G4 spectra, each mutant oligo displays a CD maximum of around 280 nm and a red-shifted minimum closer to 250 nm (Fig. S1). These features suggest a consistent departure from the parallel or hybrid topology of G-quadruplexes of the wild-type sequences. The 5.1R parallel spectra can be to an intermolecular G4 due to the guanines remaining in the mutant oligo sequence.

**Figure 2:**
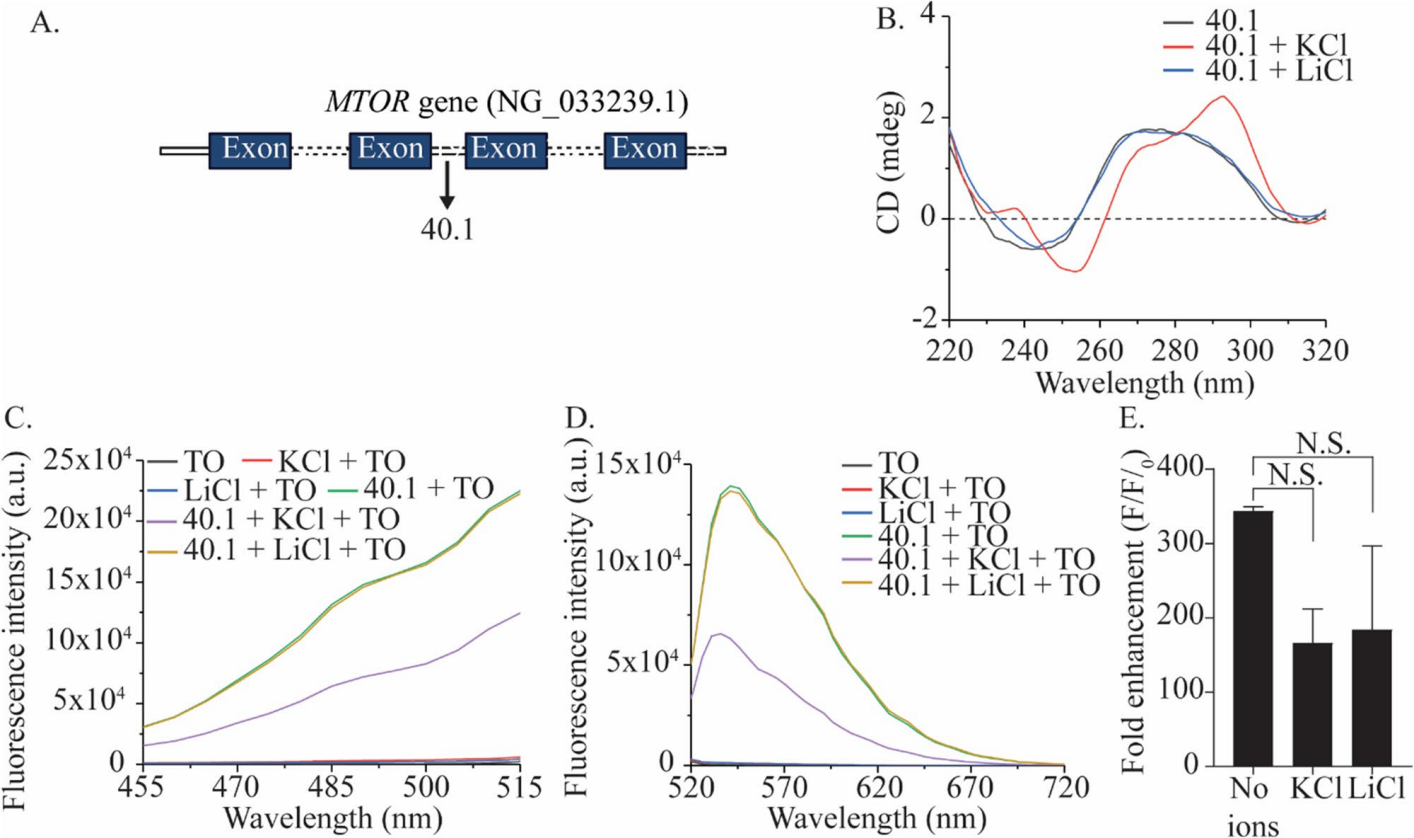
(**A**) Location of 5G sequence, 40.1, in the *MTOR* gene. (**B**) Comparative CD spectra of oligo 40.1 (5 µM) in the absence and presence of monovalent cations K^+^ and Li^+^ (100 mM). (**C**) Excitation spectra of oligo 40.1 (1 µM) in the presence and absence of K^+^ and Li^+^ and TO (2 µM) with emission captured at 534 nm. (**D**) Emission spectra of oligo 40.1 (1 µM) in the presence and absence of K^+^ and Li^+^ and TO (2 µM) when excited at 501 nm. (**E**) Fold enhancement in TO fluorescence in the presence and absence of monovalent cations, with excitation and emission at 501 and 535, respectively.

Interestingly, the QGRS mapper also indicated a putative G4 sequence consisting of 5G quartets (40.1), displaying the highest G-score of 84 starting at nucleotide 40,616 (Fig. 2A). Several studies have shown that a higher number of G’s in a G-tract and shorter loops promote stability of G4 formation^35^. Due to the unique sequence and higher G-score of this putative 5G-bearing motif, we investigated its propensity to fold into G4 structure under in vitro conditions. CD spectrum of the 40.1 sequence in absence of monovalent cations showed a characteristic maximum at ~ 265 nm and minimum at ~ 240 nm, indicating a parallel G-quadruplex structure (Fig. 2B). The effect of monovalent cations on the topology of this G4 structure was also studied. In the presence of KCl, the CD spectrum showed two maxima at ~ 290 nm and ~ 265 nm, with a minimum at ~ 240 nm, a characteristic of hybrid G4s. In the presence of LiCl, the 40.1 sequences retained the parallel G4 topology (Fig. 2B). These results indicate the possibility of an equilibrium between parallel and hybrid G4 topology of the 40.1 sequence, guided by K^+^ ions. Nevertheless, the hybrid G4 topology of 40.1 could arise from a 3 + 1 configuration or a mixed population of parallel and antiparallel conformations (2 + 2 configuration)^36^.

To further validate the G4 structure formed by the 40.1 sequence, Thiazole Orange (TO) fluorescence enhancement assay was carried out in the absence or presence of monovalent cations^37^. TO shows selective and preferential binding to stable DNA G4s via an increase in fluorescence intensity. The TO fluorescence spectra in the presence of 40.1 G4 structure showed the enhancement by ~ 330 fold (Fig. 2C,D). A decreased enhancement of TO fluorescence spectra in the presence of DNA G4 structure was observed on addition of KCl, which increased when LiCl was present instead of KCl (Fig. 2E). Together, these findings suggest that the change in topology from parallel to hybrid in the presence of K^+^ ions is accompanied by the decrease in stability of the DNA G4 structure formed by the 40.1 sequence. TO fluorescence enhancement implies suitable binding affinity of the fluorophore. Thus, it is possible that the hybrid topology of the G4 formed by 40.1 could adversely impact TO binding resulting in lower fluorescence enhancement.

### Topology of G4 structure formed in *MTOR* mRNAs

**Figure 3:**
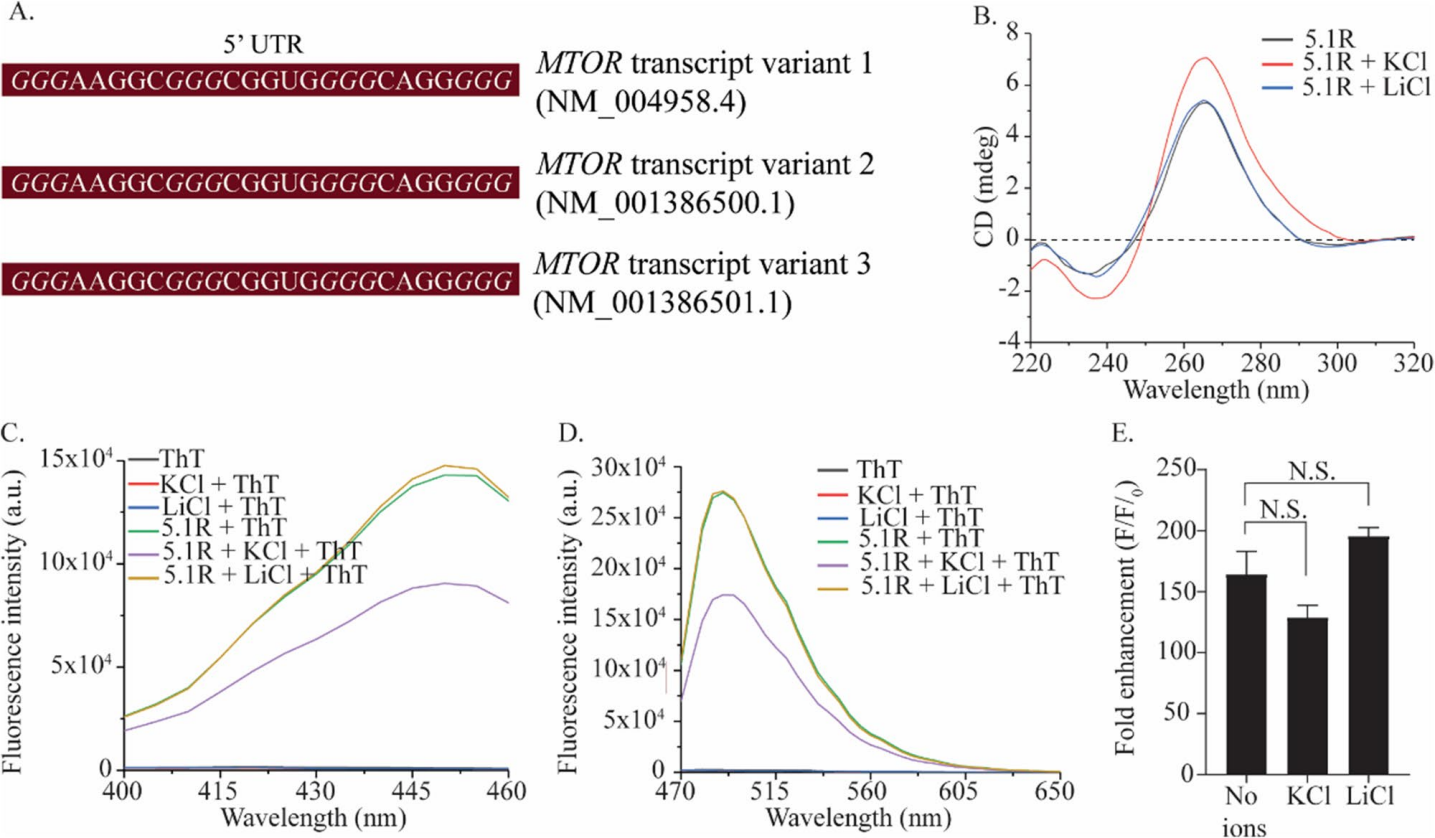
(**A**) Location of 3G sequence, 5.1R, in the three *MTOR* mRNA transcripts. (**B**) Comparative CD spectra of 5.1R (5 µM) in the absence and presence of monovalent cations K^+^ and Li^+^ (100 mM). (**C**) Excitation spectra of oligo 5.1R (2 µM) in the presence and absence of K^+^ and Li^+^ and ThT (2 µM) with emission captured at 488 nm. (**D**) Emission spectra of oligo 5.1R (2 µM) in the presence and absence of K^+^ and Li^+^ and ThT (2 µM) when excited at 445 nm. (**E**) Fold enhancement in ThT fluorescence in the presence and absence of monovalent cations, with excitation and emission at 445 and 488, respectively.

G-Quadruplex structures in RNA transcripts, especially in the UTRs, are known to affect mRNA localization, translation and several downstream processes. To examine the occurrence and distribution of G4 structures in *MTOR* mRNA, we assessed the sequences of all three functional transcript variants of *MTOR* mRNA (NM_004958.4, NM_001386500.1, NM_001386501.1) using QGRS mapper. The assessment indicated a putative G4 sequence (5.1R) at the 5′ UTR of all three transcript variants with a common and highest G-score of 41 (Fig. 3A). A nucleotide-BLAST analysis revealed that the 5.1R putative quadruplex forming sequence was conserved amongst all three transcript variants. Moreover, this sequence appears to be transcribed from the 5.1 sequence in the exonic region of *MTOR*. These findings suggest an important role of the 5.1R sequence at the 5′ UTR of *MTOR* mRNA. To investigate the propensity of the 5.1R sequence to fold into a G4 structure, 5.1R was produced by in vitro transcription from a T7 RNA polymerase promoter. CD spectra of the 5.1R sequence revealed a characteristic pattern for forming a parallel G4 topology in the presence or absence of monovalent cations. These results suggest G4 structure formation by the 5.1R sequence in *MTOR* mRNA and is consistent with the presence of parallel G4 structures in many physiologically relevant RNAs^9,11,38^. Interestingly, the topology formed by the 5.1R sequence was unaffected in the presence of monovalent cations (Fig. 3B).

To determine the stability of the G4 structure formed by the 5.1R sequence, Thioflavin T (ThT) fluorescence enhancement assay was carried out for the 5.1R sequence, in the presence or absence of monovalent cations^39^. ThT shows selective and preferential binding to RNA G4s. Similar to TO binding with DNA G4 structures, ThT showed increased fluorescence intensity upon binding with the RNA G4 structures (Fig. 3C,D). The ThT fluorescence in the presence of 5.1R G4 structure showed a ~ 160-fold enhancement. The ThT fluorescence enhancement with 5.1R was lowered in the presence of KCl while adding LiCl increased the ThT fluorescence (Fig. 3E). KCl is known to promote dimeric and intermolecular G-quadruplex formation at higher concentrations, leading to a decrease in the population of monomeric intramolecular quadruplexes. This shift could result in a decrease in TO and ThT fluorescence, as there are less binding sites for the fluorophores to bind^40,41^.

### Affinity of G4-specific ligands for *MTOR* G4 structures

**Figure 4:**
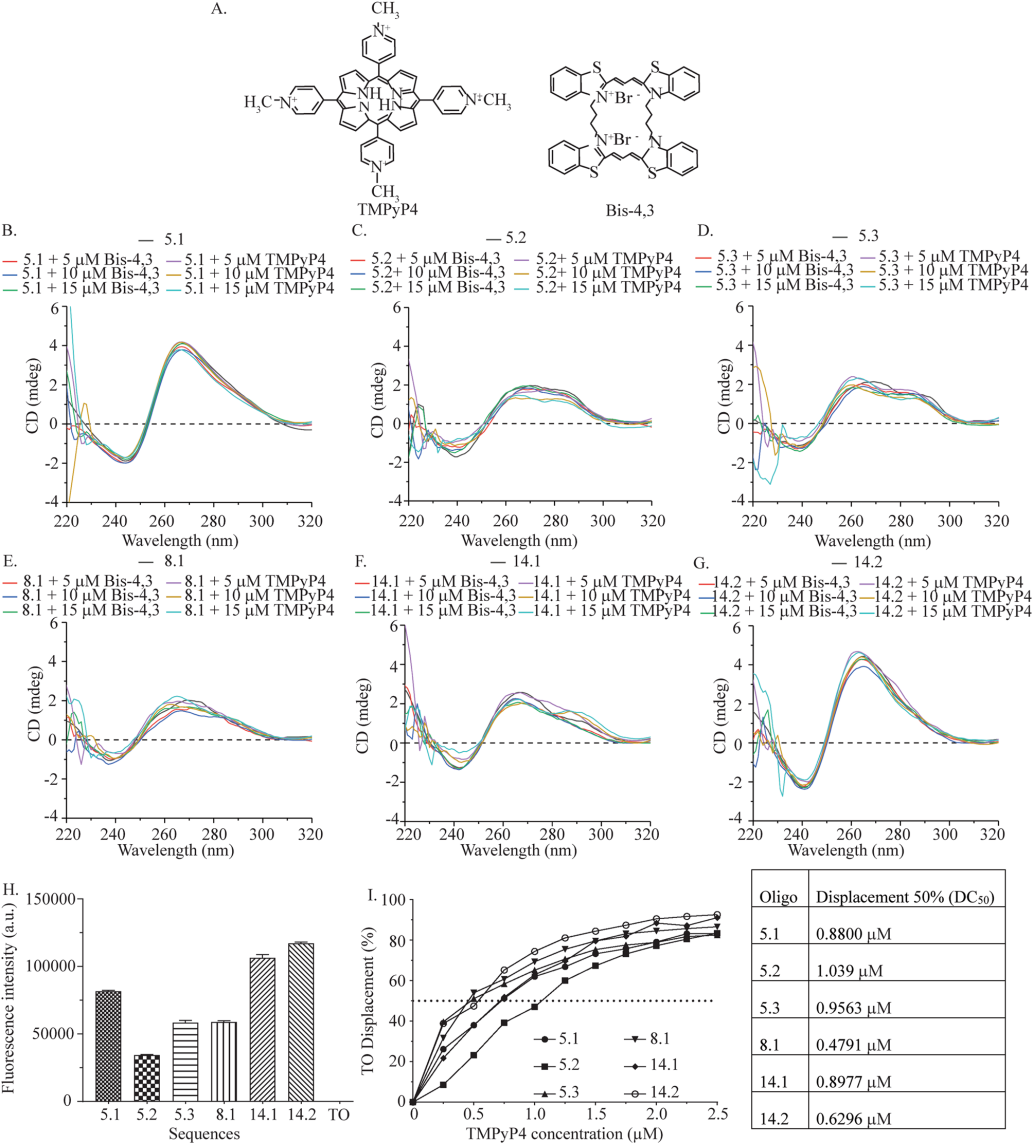
(**A**) Structure of TMPyP4 and Bis-4,3 ligands. (**B**) Comparative CD spectra of 5.1 oligo in the presence of KCl and 5, 10 and 15 μM Bis-4,3 or TMPyP4. (**C**) Comparative CD spectra of 5.2 oligo in the presence of KCl and 5, 10 and 15 μM Bis-4,3 or TMPyP4. (**D**) Comparative CD spectra of 5.3 oligo in the presence of KCl and 5, 10 and 15 μM Bis-4,3 or TMPyP4. (**E**) Comparative CD spectra of 8.1 oligo in the presence of KCl and 5, 10 and 15 μM Bis-4,3 or TMPyP4. (**F**) Comparative CD spectra of 14.1 oligo in the presence of KCl and 5, 10 and 15 μM Bis-4,3 or TMPyP4. (**G**) Comparative CD spectra of 14.2 oligo in the presence of KCl and 5, 10 and 15 μM Bis-4,3 or TMPyP4. (**H**) Fluorescence enhancement with 1 μM of *MTOR* oligos (5.1, 5.2, 5.3, 8.1, 14.1, 14.2) and 2 μM of TO. (**I**) Fluorescence intercalator displacement curves of individual *MTOR* oligos (5.1, 5.2, 5.3, 8.1, 14.1, 14.2) with TMPyP4.

The porphyrin-based ligand, TMPyP4 (Fig. 4A (left)), has been widely deployed as a G4 stabilizing ligand^8^. We have previously reported the G4-binding behaviour of a novel dimeric carbocyanine dye, Bis-4,3 (Fig. 4A (right)). Bis-4,3 displayed a strong propensity to selectively bind parallel G4 with concomitant fluorescence turn-on^42,43^. While our previous works focused on the G4-selective fluorescent reporting potential of Bis-4,3, an interest in its ligand-like behaviour led to the formulation of the current use for this ligand. We have also used Bis-4,3 to validate the effects of TMPyP4 as the two ligands have similar G4-binding sites.

CD spectroscopy can be used for probing structural perturbations of G4s caused by G4-selective ligands^44^. CD spectra of oligos 5.1, 5.2, 5.3, 8.1, 14.1, 14.2 (Fig. 4B–G) were measured in the presence of 5, 10 and 15 µM TMPyP4 and Bis-4,3. CD spectra in the presence of both ligands reveal a lack of structural distortions. CD spectra of the oligos were measured from 25 to 75 °C, at 10 °C intervals, in the presence of 10 µM TMPyP4 or Bis-4,3. In the presence of Bis-4,3, oligo 5.1 showed a decrease in the ~ 260 nm peak only at ~ 55 °C (Fig. S2A). In the presence of TMPyP4, the 260 nm peak decreased around 75 °C, indicating a stably formed G4 structure (Fig. S2B). There was a steady decrease in the 260 nm peak for oligo 8.1 in the presence of TMPyP4, and oligo 14.1 in the presence of Bis-4,3 and TMPyP4 (Fig. S2C,E,F). In contrast, the thermal spectra of oligo 8.1 with Bis-4,3 overlaid even at 75 °C, indicating a stable quadruplex (Fig. S2D). The CD experiments revealed that the binding of TMPyP4 and Bis-4,3 to the *MTOR* G4s is contingent on the oligo sequence and corresponding secondary structures.

The interaction of G-rich sequences with G4-specific ligands is now part of a canonical in vitro characterization of G-quadruplexes. Within this experimental paradigm, fluorescence displacement assays allow a straightforward comparison of the binding behaviour of putative G-quadruplexes. We used a fluorescence displacement assay to compare the binding behaviour of the porphyrin ligand TMPyP4 across the G4 sites of *MTOR*. Thiazole Orange (TO) was used as a reporter in the fluorescence displacement assay. TO has been suggested as a G4-targeting fluorescence reporter, albeit with modest quadruplex-to-duplex selectivity^45^.

The binding of TO with G4 structures results in multi-fold fluorescence enhancement. Further, G4-targeting ligands can be screened based on their ability to displace TO, resulting in a loss of fluorescence. We adapted the TO-based fluorescence displacement assay to study the behaviour of the G-rich sequences of *MTOR*. Interaction of the *MTOR* G-rich oligos with TO resulted in substantive fluorescence enhancement, especially for oligos 5.1 and 14.1 and 14.2 (Fig. 4H). We next measured the TO displacement from *MTOR* G-rich oligos as a function of TMPyP4 concentration. The binding behaviour of TMPyP4 with the G-rich *MTOR* oligos suggested comparable binding affinities. Based on the ligand concentrations corresponding to 50% of TO displacement, TMPyP4 displays a nearly identical affinity for oligos 5.1 and 14.1 (0.8800 and 0.8977 μM respectively). Interestingly, while oligo 5.3 displays a hybrid G4 topology, the binding affinity of TMPyP4 for 5.3 (0.9563 μM) is comparable to that of oligos 5.1 and 14.1 (Fig. 4I).

Primer extension assays have been used to demonstrate the presence of G4 secondary structures in a sequence of interest^46^. Based on the CD spectroscopic behaviour of the oligos in the presence of the G4 binding ligands, we conducted the primer extension assay on oligo 5.2 and 8.1 as a proof of concept for G4 formation. This experiment was performed separately with the two ligands Bis-4,3 and TMPyP4. Oligos used for this experiment are listed in Supplementary Table 2. Interestingly, oligo 5.2 and 8.1 (Fig. S3A–D) did not show a decrease in full-length product intensity. Nevertheless, the formation of truncated products in these instances indicates that the respective quadruplex structures were stabilized at the concentrations of ligands used for cellular studies. On the other hand, increasing concentrations of TMPyP4 on oligo 5.2 likely caused a shift in equilibrium from a higher order structure (viz. intermolecular quadruplex) to an intramolecular quadruplex, even in the presence of 7 M urea, an effective denaturant^47,48^ (Fig. S3E,F). This suggests that TMPyP4 has a strong influence on the structural dynamics of G-quadruplex-forming oligo 5.2. The ability of TMPyP4 to cause a shift in equilibrium in vitro could portend a variability of ligand effect under cellular conditions. These results verify the in vitro formation of G4s by G-rich regions in the regulatory region of the *MTOR* gene. In the cellular context, this could lead to truncated *MTOR* transcripts and consequent reduced mTOR protein levels. Thus, these results highlight the possibility of using ligand-mediated G4-stabilization to regulate mTOR protein levels.

### Influence of G4-targeting ligands on mTOR activity in HeLa and SHSY-5Y cells

**Figure 5:**
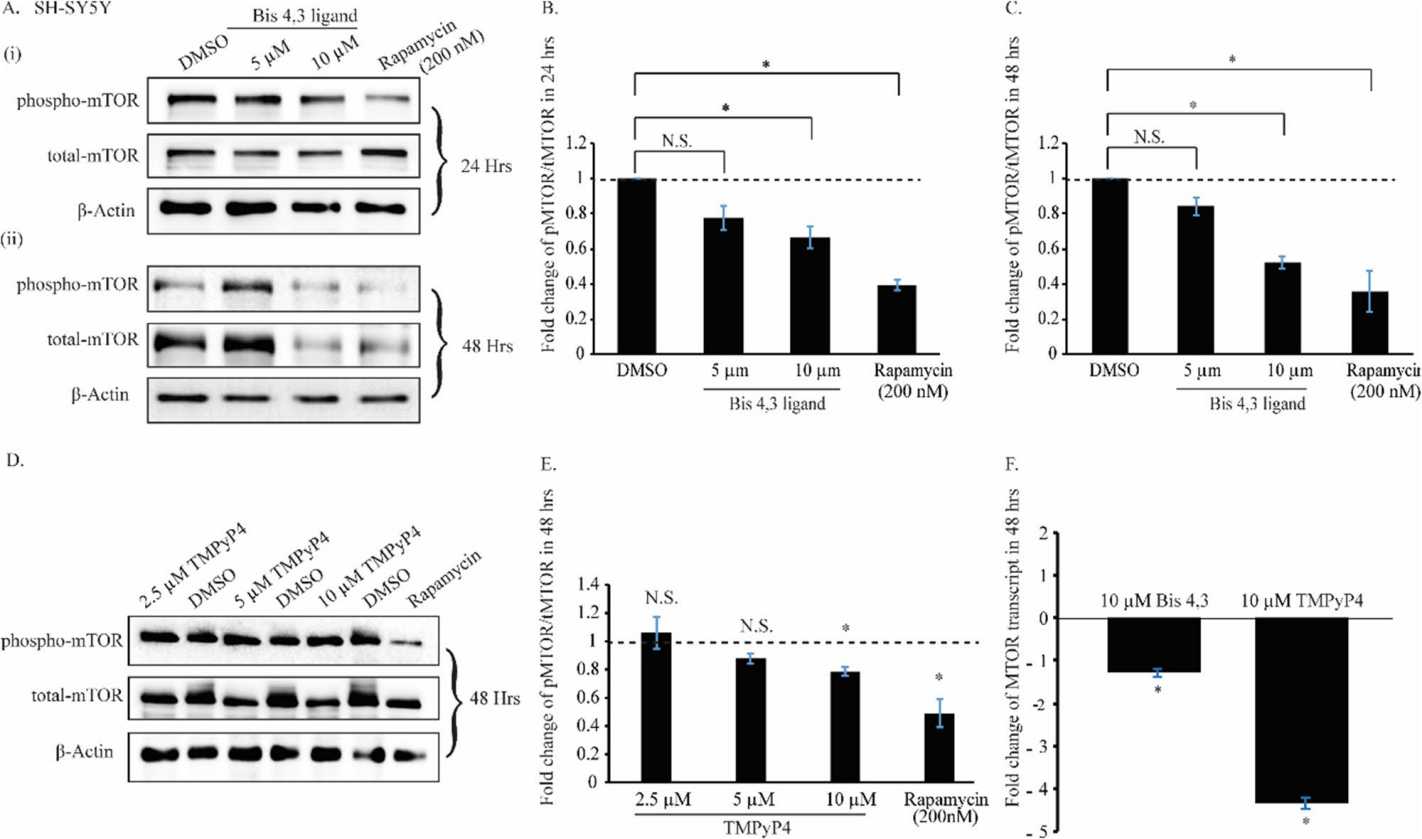
(**A**) Western blot of phospho-mTOR and total mTOR level in SHSY-5Y cells after Bis-4,3 treatment for (**i**) 24 h and (**ii**) 48 h. Rapamycin treatment was used as a positive control. (**B**) graphical analysis of the phospho/total mTOR levels of SHSY-5Y cells after Bis-4,3 treatment for 24 h. (**C**) Graphical analysis of the phospho/total mTOR levels of SHSY-5Y cells after Bis-4,3 treatment for 48 h. (**D**) Western Blot of phospho-mTOR and total mTOR level in SHSY-5Y cells after TMPyP4 treatment for 48 h. Rapamycin treatment was used as a positive control. (**E**) Graphical analysis of the phospho/total mTOR levels of SHSY-5Y cells after TMPyP4 treatment for 48 h. (**F**) Graphical representation of qRT-PCR ΔΔCT values of mTOR fold change for both Bis-4,3 and TMPyP4 treated condition in SHSY-5Y cells for 48 h compared to respective DMSO treated condition.

The presence of G4 structures in the *MTOR* gene that are amenable to interaction with G4-ligands led us to probe the functional relevance of these structures in the cells. We tested the effects of G4-selective ligands on endogenous mTOR protein levels in HeLa and SHSY-5Y cell lines by western blotting. We first optimized the ligand effect of Bis-4,3 towards the expression of mTOR protein as indicated by western blots in HeLa and SHSY-5Y cells. 5 and 10 µM of Bis-4,3 treatment for 24 h was accompanied by no significant change in mTOR activity. mTOR activity was measured via the ratio of phospho mTOR/total mTOR levels in SHSY-5Y cells (Fig. 5A(i),B) and HeLa cells (Fig. S4A(i),B). Notably, 10 µM treatment of Bis-4,3 for 48 h significantly downregulated mTOR activity levels in both cell lines by 1.9 and 1.5 folds, respectively (Fig. 5A(ii),C) and (Fig. S4A(ii),C). Considering the use of DMSO as a solvent for the ligands, an equal volume of DMSO was used as a control for the experiments and the mTOR inhibitor rapamycin (200 nM) was used as a positive control. Treatment with rapamycin for 48 h significantly downregulated the mTOR activity by 3 and 2.8-fold in SHSY-5Y and HeLa cells, respectively.

Following the preliminary assessment of G4-selective ligand effects on mTOR activity, optimization of ligand concentrations was pursued by using variable ligand concentrations across both cell lines. The optimum concentration of TMPyP4 was evaluated by using different concentrations of the ligand (2.5 µM, 5 µM, and 10 µM) for 48 h in HeLa (Fig. S4D,E) and SHSY-5Y cells (Fig. 5D,E). Based on western blot analysis, 10 μM of TMPyP4 downregulated the mTOR activity in HeLa and SHSY-5Y cells by 1.4 folds and 1.27 folds, respectively, compared to the DMSO control.

Next, mTOR transcript levels were measured in the presence of Bis-4,3 (10 μM) and TMPyP4 (10 μM) for 48 h in HeLa (Fig. S4F) and SHSY-5Y (Fig. 5F). The qRT-PCR data indicated significant downregulation of *MTOR* transcript levels by 3 and 4-fold for HeLa and SHSY-5Y cells, respectively. The downregulated mTOR activity in both cell lines corroborated our hypothesis that the G4-stabilization of the *MTOR* gene is capable of impeding the expression of the mTOR protein.

### G4 targeting ligands and induction of autophagy in HeLa and SHSY-5Y cells

**Figure 6:**
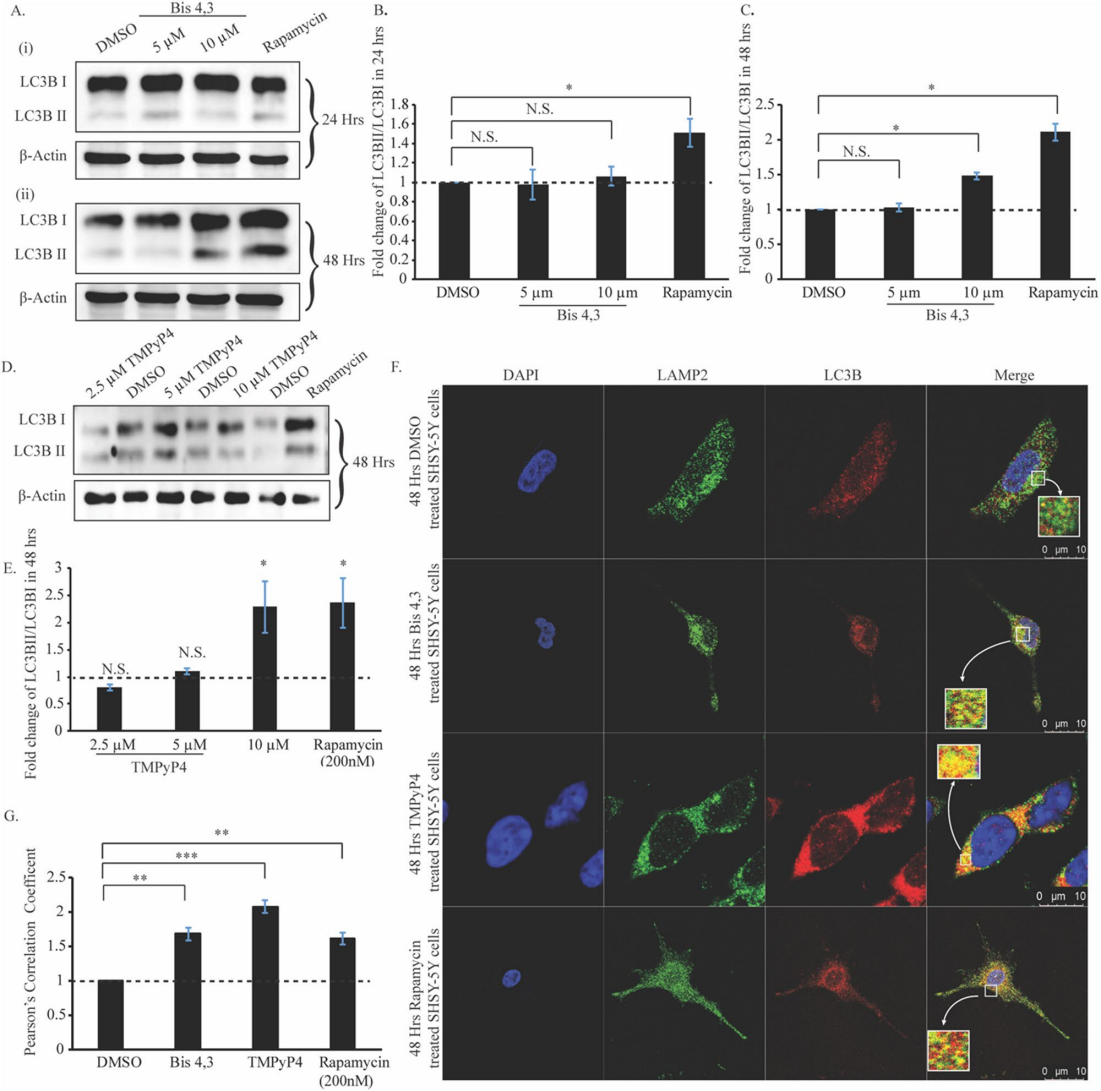
(**A**) Western Blot of LC3B level in SHSY-5Y cells after Bis-4,3 treatment for (**i**) 24 h and (**ii**) 48 h. Rapamycin treatment was observed as a positive control. (**B**) Graphical analysis of the LC3BII/LC3BI levels of HeLa cells after Bis-4,3 treatment for 24 h. (**C**) Graphical analysis of the LC3BII/LC3BI levels of HeLa cells after Bis-4,3 treatment for 48 h. (**D**) Western Blot of LC3B level in HeLa cells after TMPyP4 treatment for 48 h. Rapamycin treatment was observed as a positive control. (**E**) Graphical analysis of the LC3BII/LC3BI levels of HeLa cells after TMPyP4 treatment for 48 h. (**F**) Confocal images of DMSO, Bis-4,3, TMPyP4 and rapamycin-treated HeLa cells. LAMP2 and LC3B proteins are indicated in green and red, respectively. The nucleus was stained with DAPI. (**G**) Graphical representation of Pearson’s correlation coefficient for the co-localization.

The mTOR protein is a potent regulator of several metabolic pathways in the cell^17^. One such catabolic pathway is autophagy, where the cell tries to sustain or maintain homeostasis by auto-digesting aberrant or unwanted proteins, lipids and cytosolic organelles under stress or starved conditions. We studied the effect of G4 stabilizing ligands, Bis-4,3 and TMPyP4, on autophagy induction, in an attempt to elucidate the involvement of G4 motifs of *MTOR*. It is widely known that LC3B is the marker for autophagy induction^49^. An increased ratio between the lipidated (LC3BII) and cytosolic LC3B (LC3BI) signifies upregulation of autophagy compared to the basal level. First, the optimum ligand concentrations and incubation times for application of Bis-4,3 in SHSY-5Y cells were ascertained. As evident from the western blots, no significant change was observed in LC3BII levels compared to LC3BI levels upon 24-h treatment with Bis-4,3 ligand (Fig. 6A(i),B). However, treatment with 10 μM concentration of Bis-4,3 for 48 h upregulated the LC3BII/LC3BI levels by 1.47-fold (Fig. 6A(ii),C). Next, the optimum parameters for treatment with TMPyP4 were determined analogously. We observed significant upregulation of LC3BII/LC3BI levels upon treatment with 10 μM (2.3-fold) TMPyP4 for 48 h (Fig. 6D,E).

Subsequently, we verified the autophagic flux by studying the fusion of autophagosomes with lysosomes via confocal microscopy. Verification of autophagy maturation or autophagic flux is essential, as the accumulation of autophagosomes may not necessarily indicate induction of autophagy. The fusion between autophagosomes and lysosomes is considered a substantive evidence of autophagy induction^50^. Co-localization of an autophagosomal marker, LC3B and LAMP2A, a lysosomal marker suggests that autophagosomes efficiently co-localize with lysosomes in SHSY-5Y cells treated with the G4 stabilizing ligands Bis-4,3 and TMPyP4 (Fig. 6F). The extent of co-localization was measured by Pearson’s correlation coefficient using the Jacop plug-in in ImageJ software (Fig. 6G).

The LCBII/LC3BI levels were similarly determined for HeLa cells treated with both G4 targeting ligands. Treatment with Bis-4,3 for 24 h was not accompanied by significant alterations in LC3B ratios (Fig. S5A(i),B). However, treatment with 10 μM of the ligand for 48 h led to an elevated ratio by 1.2-fold (Fig. S5A(ii),C). Conversely, treatment of HeLa cells with 10 μM of TMPyP4 for 48 h substantially upregulated the LCBII/LC3BI levels by 2-fold compared to the control DMSO treated condition (Fig. S5D,E). Confocal microscopy revealed autophagic flux for both Bis-4,3 and TMPyP4 treated HeLa cells based on the co-localization between LC3B and LAMP2A (Fig. S5F). Co-localization coefficient was measured by Pearson’s correlation coefficient (Fig. S5G). CellRox Deep Red reagent was also used to determine whether ligand treatment led to autophagy induction via intracellular ROS production or the MTOR G4 DNA axis. No significant change was observed with 10 μM Bis-4,3 and TMPyP4 treatment for 48 h as compared to the DMSO (vehicle control). Therefore, it is unlikely that the cellular ROS production led to autophagy induction (Fig. S6).

**Figure 7:**
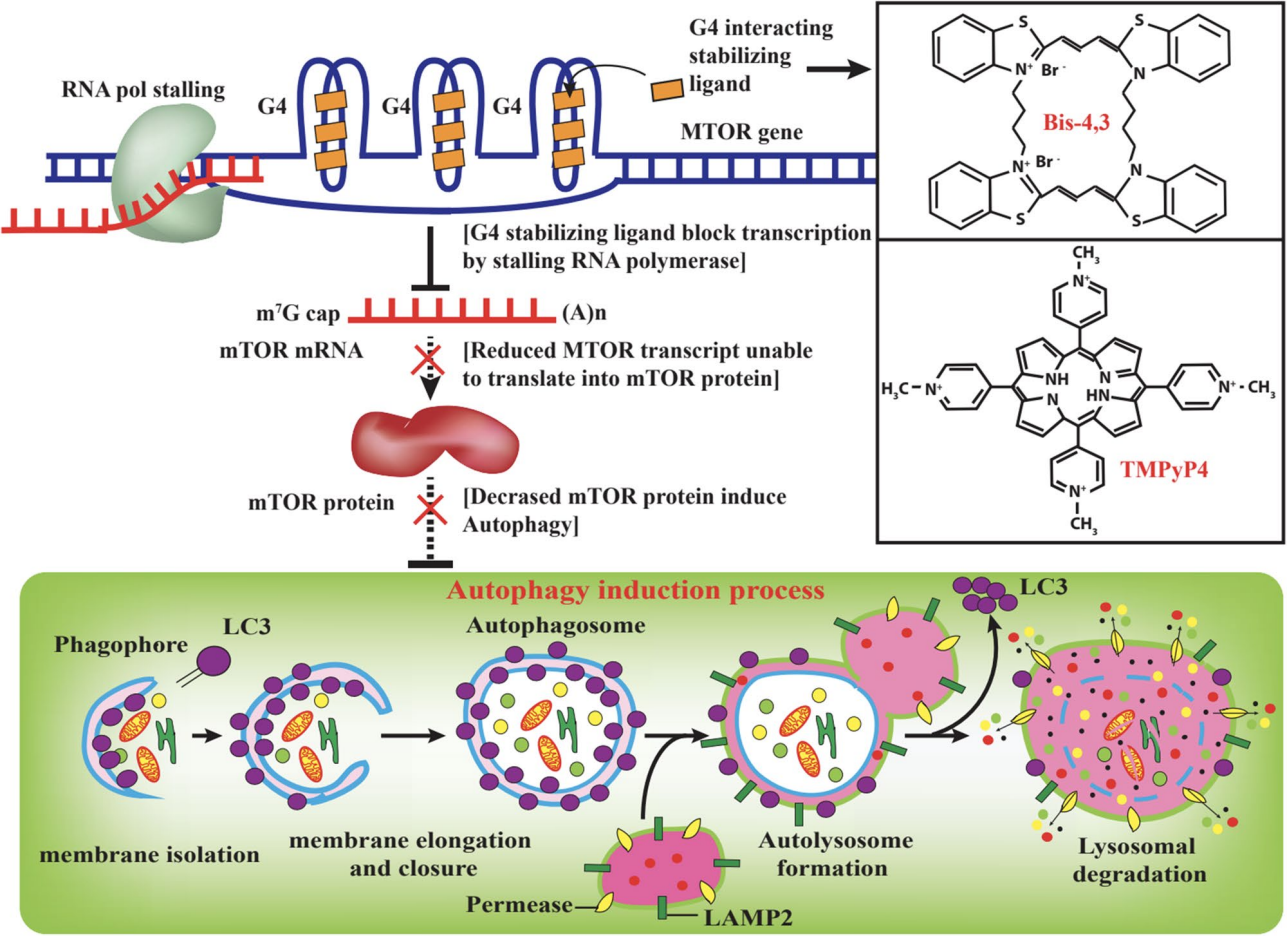
Putative mechanism of *MTOR* G4 stabilization and autophagy induction.

Our results hint that mTOR protein deficiency due to the effect of G4-stabilizing ligands leads to upregulation of the LC3BII/LC3BI levels, an established autophagy induction marker. Moreover, the co-localization between LC3B and LAMP2A implicated the autophagic flux via increased LC3B levels. This observation precludes LC3B elevation due to blockage of autophagosome and lysosome fusion. Overall, the current work highlights the putative molecular mechanism of G4 motifs in the *MTOR* gene that can potentially be leveraged toward induction of autophagy using G4 stabilizing ligands (Fig. 7). Other possible G4 gene targets as well as MTOR dependent, independent targets that could lead to or prevent autophagy as a result of TMPyP4 and Bis-4,3 treatment are yet to be identified.

## Conclusions

G4 ligand induced autophagy can be a function of several pathways including cellular stress responses or lysosomal drug sequestration resulting in lysosomal membrane permeabilization^51^. For example, treatment of cervical cancer and osteosarcoma cells with G4 ligand 20A resulted in an enrichment of RNA and proteins involved in the lysosomal pathway accompanied by MTOR inhibition, global DNA damage and activation of autophagy that causes onset of senescence and protects the cell from cell death^16,52^. Treatment of osteosarcoma cells with CX-5461, a G4 ligand, lead to induction of autophagic cell death caused by MTOR inhibition, activation of AMPK pathway and increased levels of p53, SESN1, SESN2 and CDKN1A^53^. Alternatively, G4 ligands may also induce autophagy by interacting with DNA and RNA G4 motifs of genes involved in the autophagic and lysosomal pathway and regulating their expression levels. While the previous studies have reported ligand induced autophagic induction and reduced mTOR protein levels, the contribution and effect of the G4s in the MTOR gene have not been taken into account and are novel. There are no previously reported effects of TMPyP4 and Bis-4,3 on LC3BII/LC3B1 ratios and mTOR expression levels. To this end, the present study has led to the following findings: (i) Several regions of the *MTOR* gene have the propensity to form G-quadruplex secondary structures that are receptive to G4-targeting ligands; (ii) All three *MTOR* mRNA transcripts harbour a parallel RNA G-quadruplex and, (iii) G4-ligand treatment results in induction of autophagy in HeLa and SHSY-5Y cells. While we observe an induction of autophagy upon use of two different G4 ligands, parallel MTOR dependent or independent pathways could also be contributing to the observed effect. Thus, in spite of our identification of G4 motifs in the *MTOR* gene and the manifestation of an apparent G4 ligand effect, we are not claiming exclusive targeting of MTOR G4s by the ligands.

It was recently demonstrated that PDS and BRACO-19 induced stabilization of G4-DNA structures in the *Atg7* gene leads to downregulation of autophagy in neurons^54^. While these may be contradictory to our reports, the observed induction of autophagy upon treatment with Bis-4,3 and TMPyP4 could likely be a result of the combination of the *MTOR* G4 structures, ligands and cell lines used in this study, as well as other targets that are yet to be identified. Further attempts can be made to determine synergistic effects (such as role of oncogenes namely *KRAS* and *c-Myc*) that could lead to this observed effect of autophagy by Bis-4,3 and TMPyP4, both promiscuous G4 targeting ligands. Future avenues of study could include identification of parallel pathways regulated by these ligands in HeLa, SHSY-5Y cell lines, effect of the 5.1 RNA G4 on translational regulation and ligand mediated alternative splicing. Correlation between exonic quadruplex within the transcript of *MTOR* gene and the details pertaining to the relative importance of various G4-sites in the other regulatory regions of *MTOR* are currently being worked on in our laboratory.

## Methods

### Oligonucleotides and chemicals

The oligonucleotides used in the current work are listed in Supplementary Table 1 and were purchased either from Sigma Aldrich, USA or IDT in lyophilized form. Nuclease-free water was used to prepare stocks of 100 µM. Rapamycin (S1039) from Selleck chemical LLC and TMPyP4-tosylate (4253) from Tocris were procured without further purification. Bis-4,3 was provided as a gift by Dr Prathap Reddy Patlolla of IIT Gandhinagar.

### DNA and RNA G4 in silico analysis for identifying putative quadruplex-forming sequences

The QGRS mapper tool was used to identify putative quadruplex forming sequences (PQS) in the *MTOR* gene and RNA using the parameters as follows: Maximum length: 45, Min. G-tract: 2, Loop size: 0–10.

### In vitro transcription

The DNA oligonucleotides required for in vitro transcription were ordered from Sigma-Aldrich, USA. 5.1R sequence was transcribed from the T7 RNA promoter using the HiScribe™ T7 High Yield RNA Synthesis Kit (New England Biolabs, USA), following the manufacturer’s protocol. The DNA oligonucleotides in the transcribed RNAs solution were digested using DNase I, RNase-free (Thermo Fisher Scientific, USA) and the RNAs were cleaned up and eluted using Monarch^®^ RNA Cleanup Kit (500 μg) (New England Biolabs, USA), following manufacturer’s protocol. The concentration and purity of eluted RNAs were quantified using NanoDrop™ 2000 spectrophotometer (Thermo Fisher Scientific, USA) and were stored at − 80 °C until further use.

### Circular dichroism spectroscopy

Samples containing 5 μM DNA and RNA were folded in a buffer containing 10 mM Tris–Cl (pH 7.5) and 10 mM Tris–Cl (pH 7.5), 0.01 mM EDTA (pH 8.0), respectively, by incubating at 95 °C for 5 min followed by gradually cooling to room temperature over 2 h. DNA and RNA samples were supplemented with 100 mM KCl or LiCl or 5, 10, 15 μM of either TMPyP4 or Bis-4,3, while folding. CD spectra were recorded on a JASCO J-815 spectropolarimeter using a quartz cell of 1 mm optical path length and an instrument scanning speed of 100 nm/min over a wavelength range of 190–320 nm. For temperature-based CD studies, samples were prepared as above and incubated at 25–75 °C at intervals of 10 °C/h. The reported spectra of each sample represents an average of 3 scans and is baseline-corrected.

### TO and ThT fluorescence enhancement assays

Fluorescence enhancement assays were performed using Thiazole Orange (TO) and Thioflavin T (ThT) (Sigma-Aldrich, USA) as a DNA and RNA G4 binding dye, respectively, in a 96-well black fluorescence microplate. DNA samples (1 µM) were folded in 10 mM Tris–Cl (pH 7.5) in supplementation with 100 mM KCl or LiCl, while RNA samples (2 µM) were folded in 10 mM Tris–Cl (pH 7.5) and 0.01 mM EDTA (pH 8.0) in supplementation with 100 mM KCl or LiCl, by incubating at 95 °C for 5 min followed by gradually cooling to room temperature over 2 h. TO and ThT (2 μM) were added to the folded DNA and RNA G4, respectively. TO excitation spectra were obtained, with emission captured at 534 nm, while the emission spectra were obtained after excitation at 534 nm. Similarly, ThT excitation spectra were obtained with emission captures at 488 nm, while the emission spectra were obtained after excitation at 445 nm. Single-point fluorescence intensities were also obtained for TO and ThT at the mentioned wavelengths. The fluorescence of samples was measured at 25 °C using Envision XCite Multimode Microplate Reader (PerkinElmer, USA).

### G4-FID

Experiments were carried out in 96-well black microplates. Each well consists of (a) pre-folded oligonucleotide with TO and (b) a freshly prepared ligand solution. Gradient ligand concentration (0, 0.125, 0.25, 0.375, 0.5, 0.625, 0.75, 1.0, 1.25, 1.5, 2.0 and 2.5 μM) was used in the experiment. After orbital mixing, the fluorescence was measured (501/532). The percentage TO displacement was calculated as previously reported^45^. The affinity of the ligands with a particular oligonucleotide was estimated by DC_50_ values, which is the ligand concentration at which half of the fluorescence displacement was observed.

### Polymerase extension assay

Primers used in the current work are listed in Supplementary Table 2. Nuclease-free water was used to prepare 100 µM solutions. Briefly, primers were mixed with respective template DNA in an annealing buffer (50 mM Tris pH 8.0, 10 mM MgCl_2_) and denatured by heating at 95 °C for 5 min before cooling to room temperature over 2 h. 1 × Polymerase extension buffer (40 mM Tris HCl pH 8.0, 1 mM MgCl_2_, 5 mM DTT, 100 μg/ml BSA, 250 μM ATP and 0.1% NP40), 1 M KCl, 2 mM dNTPs were added to the mixture with either 0–20 μM TMPyP4 or Bis-4,3 and incubated at 37 °C for 1 h. 1 U/μl per reaction of *Taq* DNA polymerase (Thermo Fisher Scientific) was added to the mix and heated at 40 °C for 1 h. An equal volume of stop buffer (95% Formamide, 0.05% Bromophenol Blue, 20 mM EDTA, 0.05% Xylene cyanol) was added to stop the reaction. The products were separated on a 15% denaturing (UREA) polyacrylamide gel, visualized on Syngene GBOX gel doc system or ChemiDoc™ MP Imaging system using Diamond™ Nucleic Acid dye (Promega Corporation), and band intensities were quantified using ImageJ software.

### Cell culture

Human neuroblastoma, SHSY-5Y cells, (obtained from National Cell Science Centre, Pune, India) were cultured in DMEM-F12 (Gibco) supplemented with 10% fetal bovine serum (Gibco) at 37 °C in a 5% CO_2_ atmosphere under humidified condition. Similarly, HeLa cells were cultured in DMEM (Gibco) supplemented with 10% fetal bovine serum (Gibco) at 37 °C in a 5% CO_2_ atmosphere under humidified conditions.

### Cell lysate preparation from mammalian cells

Phosphate buffer saline (PBS) washed pellets from cell lines were lysed on ice in lysis buffer (1 M Tris–HCl, pH 7.5, 1 N NaCl, 0.5 M EDTA, 1 M NaF, 1 M Na_3_VO_4_, 10% SDS, 20 mM PMSF, 10% Triton X-100, 50% glycerol) for 30 min in the presence of complete protease inhibitor (Roche Diagnostics) and centrifuged at 13,000*g* for 15 min. Protein concentration of the supernatant was calculated by the Bradford protein estimation assay.

### Western blot

The cell lysate (25 μg) was separated on SDS PAGE and transferred to a 0.22 μm PVDF membrane (Millipore Corporation), which was blocked by 5% skimmed milk in TBST (50 mM Tris–HCl, 150 mM NaCl, pH 7.5 containing 0.05% Tween 20). The membrane was incubated with HRP conjugated secondary antibody after primary antibody incubation. Blots were developed with an ECL kit (Clarity Western ECL substrate kit, BioRad). Quantification of western blots was carried out using Quantity One software of Bio-Rad. At least three separate experiments were analyzed, and band intensities were normalized to loading control. p-values were determined using an unpaired *t*-test.

### Antibodies

Antibodies details are as follows. β-Actin (Abcam, ab8226), Phospho-mTOR (CST, 5536S), mTOR (CST, 2983S), LC3B (Novus Biologicals, NBP2-46892).

### Immunocytochemistry

SHSY-5Y and HeLa cells were grown on Nunc™ glass bottom dishes and allowed to reach 70–80% confluency. For immunostaining, cells were first washed with PBS once, fixed with 4% formaldehyde for 20 min and washed again to remove residual formaldehyde. Cells were then permeabilized using 0.1% saponin (Sigma-Aldrich, USA) for 10 min. Samples were subsequently blocked with 10% FBS in PBS for 60 min. After a PBS wash, the cells were incubated overnight with the primary antibody at 4 °C. The samples were washed with PBS and incubated with the secondary antibody for 60 min at room temperature. Samples were imaged using Laser Scanning Confocal microscopy (Leica SPI8) using the 63 × oil immersion objective at 405 nm, 488 nm and 594 nm wavelengths.

### RNA isolation, cDNA preparations and real-time PCR

RNA was isolated from both SHSY-5Y and HeLa cells by TRIzol Reagent (Invitrogen, USA) following the manufacturer’s protocol and quantified using Nanodrop 2000 spectrophotometer (Thermo Scientific, USA). 2 μg RNA was used to synthesize the first strand of cDNA using oligodT primers (Fermentas) and reverse transcriptase (Invitrogen). RT-PCR reaction was carried out using 2× SyBr green Universal PCR Master Mix (Applied Biosystems, USA) in ABI Prism 7500 Real time PCR system. Primer sequences are mTOR Fw: 5′ AATGAGAGGAAAGGTGGCATCTTGGC 3′; mTOR Rv: 5′ GGTCAGCACCCAGCCATTCCAGGGCT 3′. The absolute quantification given by the software was in terms of CT values. The relative quantification of target genes was obtained by normalizing with the internal control gene (GAPDH gene).

### ROS measurement

Intracellular ROS generation was assayed as described in the CellROX^®^ Deep Red assay kit (Invitrogen™, USA). Briefly, adhered HeLa cells were treated with 10 μM Bis-4,3 and TMPyP4 for 48 h. CellRox^®^ DeepRed reagent was added to a final concentration of 5 μM and incubated at 37 °C for 30 min. The medium was removed and cells were washed thrice with PBS before fluorescence intensity was measured in Envision XCite Multimode Microplate Reader (PerkinElmer, USA) at 640/665 nm (Excitation/emission). The fluorescence intensity of CellROX Deep Red reflects the ROS levels. Statistical significance was calculated using an unpaired *t*-test.

### Statistical analysis

The mean and standard deviation were calculated by Microsoft office. For statistical analysis, an unpaired *t*-test was carried out. Statistical significance is shown with asterisks: *p ≤ 0.05; **p ≤ 0.001, ***p ≤ 0.0001.

### Supplementary Information

Supplementary Information.

## Data Availability

All data generated or analysed during this study are included in this published article (and its supplementary information files).
