# Supplementary material for: G-quadruplexes in MTOR and induction of autophagy
Source: Sci Rep. 2024 Jan 30;14:2525. doi: 10.1038/s41598-024-52561-y (PMC10827794; doi:10.1038/s41598-024-52561-y)

Supporting Information

G-quadruplexes in MTOR and induction of autophagy

Piyali Majumder[a], Chinmayee Shukla[a], Arjun Arya[a], Shubham Sharma[a] and Bhaskar Datta[*a,b]

[a]Department of Biological Engineering, Indian Institute of Technology Gandhinagar, Palaj, Gandhinagar - 382355, Gujarat, India

[b]Department of Chemistry, Indian Institute of Technology Gandhinagar, Palaj, Gandhinagar - 382355, Gujarat, India

Supplementary Table 1

Oligonucleotides	Sequence
**WT_5.1**	5'TAGAGGACAGCGGGGAAGGCGGGCGGTGGGGCAGGGGGCCTGAAGCGGCGGTACCGGTGCTGGCGGCGGCAGCTGAGGCC
**MT_5.1**	TAGAGTACAGCGTGTAAGTCGTGCGTTGTGTCAGTGTGCCTGAAGCGTCGTTACCGTTGCTGTCGTCGTCAGCTGAGTCC
**WT_5.2**	CCGCGCGGTGAGTCTAGGGCCTGGCACGACCCCTCTAGGGCGGCGTAATGTCCAGACCCA
**MT_5.2**	CCGCGCGTTGAGTCTAGTGCCTGTCACGACCCCTCTAGTGCGTCGTAATGTCCAGACCCA
**WT_5.3**	CGGCCAGCCTGCCCGTGGGGGGGCCAGGGGAAGCCGCCCGTCTGGGACGTGGGGTCCCCA
**MT_5.3**	CGTCCAGCCTGCCCGTGTGTGTGCCAGTGTAAGCCGCCCGTCTGTGACGTGTGTTCCCCA
**WT_8.1**	CTGCCTGACAAGGGCTAGGGGGAGATCTGTGTTGGGTGACCAGGGCCATA
**MT_8.1**	CTGCCTGACAAGTGCTAGTGTGAGATCTGTGTTGTGTGACCAGTGCCATA
**WT_14.1**	AAAGGGAAGTATGGCTGGTGTACCGGGAGCAAGGGAGAGGGAAGGAGGGGCCAAATTAATAGGCCCACGGT
**MT_14.1**	AAAGTGAAGTATGTCTGTTGTACCGTGAGCAAGTGAGAGTGAAGTAGGTGCCAAATTAATAGTCCCACGTT
**WT_14.2**	ATAGAGAGTGATGGGGAGGGGGTGAGTGGAGGGTCAAGGAAGGCCCTCCCTGAGGTGGTGTTATTTGACGGGGGTCCACA
**MT_14.2**	ATAGAGAGTGATGTGTAGTGTGTGAGTGTAGTGTCAAGTAAGTCCCTCCCTGAGTTGTTGTTATTTGACGTGTGTCCACA
**WT_40.1**	GGGGGCGGGGGCGGGGGTGGGGGGAAGAAAAATTCCTAGCTGTAGCTGACTGCA
**MT_40.1**	GTTTGCGTTTGCGTTTGTGTTTGGAAGAAAAATTCCTAGCTGTAGCTGACTGCA
**T7 promoter sequence for WT_5.1R and MT_5.1R**	TAATACGACTCACTATAGCGAAA
**WT_5.1R_antisense DNA template**	TGCAGTCAGCTACAGCTAGGCCGCTTCAGGCCCCCTGCCCCACCGCCCGCCTTCCCCTTTCGCTATAGTGAGTCGTATTA
**WT_5.1R (RNA obtained after IVT and clean up)**	GCGAAAGGGGAAGGCGGGCGGUGGGGCAGGGGGCCUGAAGCGGCCUAGCUGUAGCUGACUGCA
**MT_5.1R_antisensense DNA template**	TGCAGTCAGCTACAGCTAGGCCGCTTCAGGCACACTGACACAACGCACGACTTACACTTTCGCTATAGTGAGTCGTATTA
**MT_5.1R (RNA obtained after IVT and clean up)**	GCGAAAGUGUAAGUCGUGCGUUGUGUCAGUGUGCCUGAAGCGGCCUAGCUGUAGCUGACUGCA

Supplementary Table 2

The sequences of the oligonucleotides used for polymerase stop assay

oligonucleotides	Sequence
**Template_5.2**	**5’** CCGCGCGGTGAGTCTAGGGCCTGGCACGACCCCTCTAGGGCGGCGTAATGTCCAGACCCA **3’**
**5.2 Primers**	**5’** CGTGGGTCTGGACAT **3’**
**Template_8.1**	**5’** CTGCCTGACAAGGGCTAGGGGGAGATCTGTGTTGGGTGACCAGGGCCATAAGTAAATTTCATCATACT **3’**
**8.1 Primers**	**5’** AGTATGATGAAATTTACTTATGG **3’**

Supplementary Figures

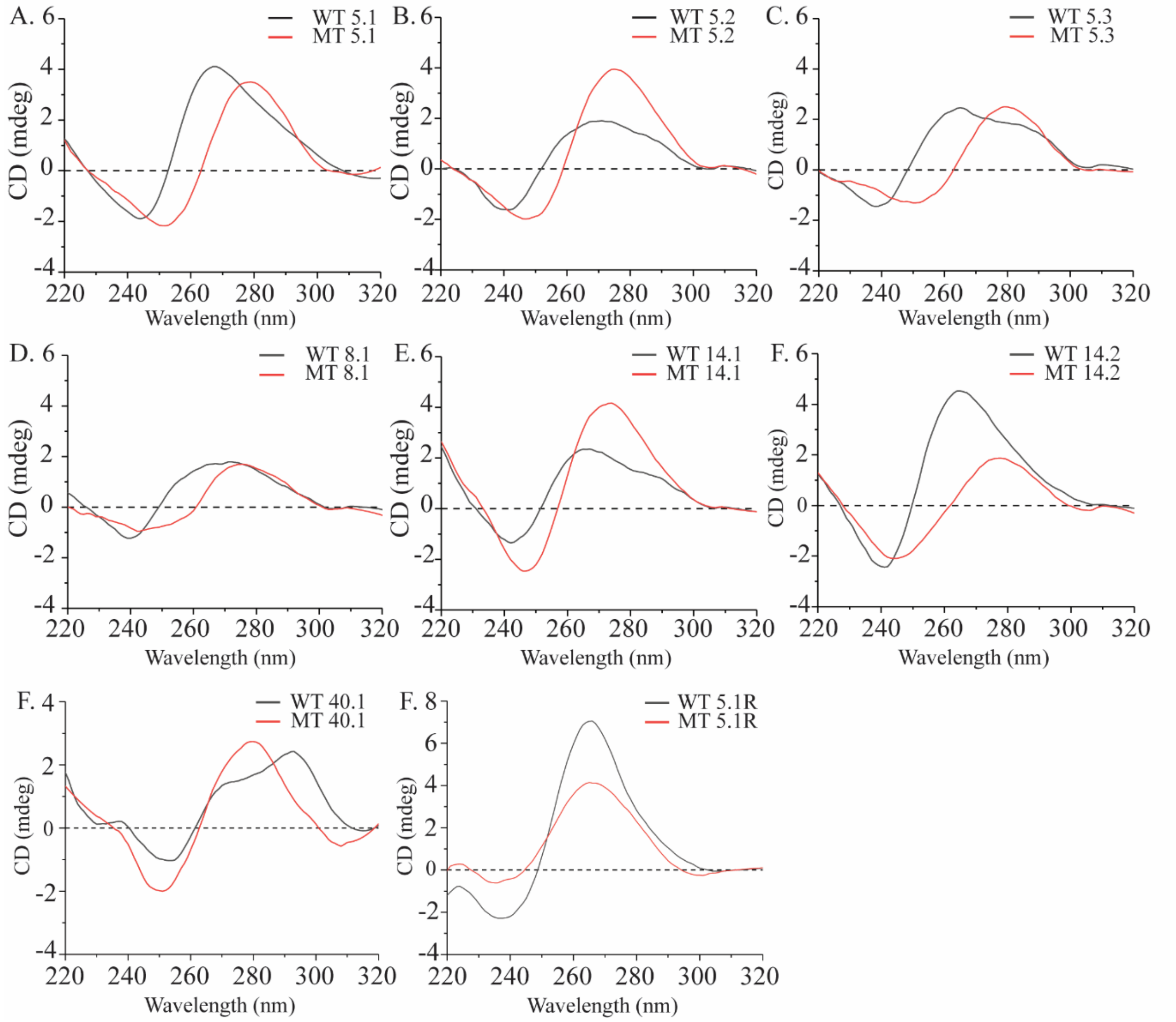

Figure S1. The CD spectra for all wild type (black) and mutant (red) oligos (5.1, 5.2, 5.3, 8.1, 14.1, 14.2, 40.1 and 5.1R) in presence of KCl

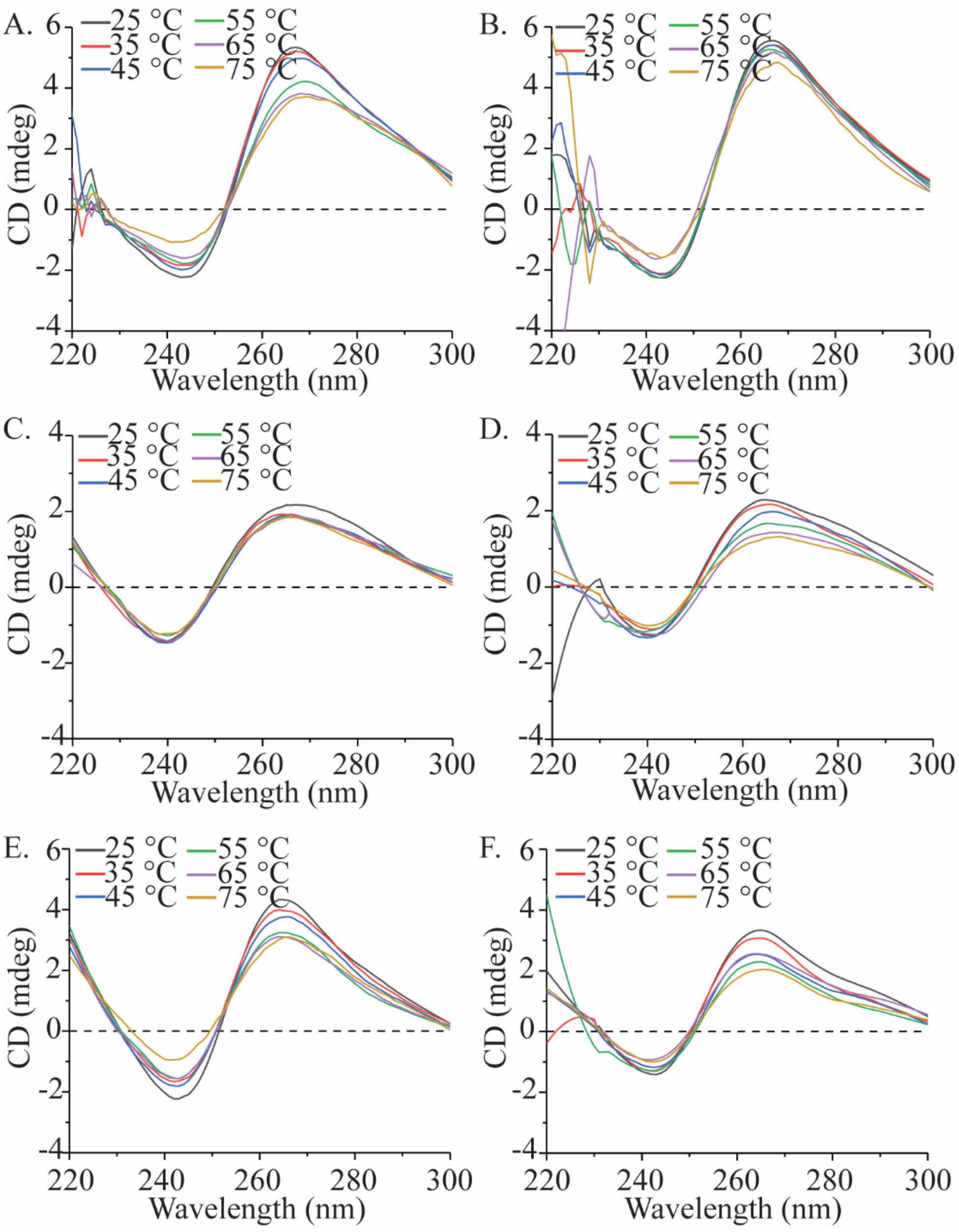

Figure S2. A, The temperature dependent (25, 35, 45, 55, and 65°C) CD spectra of 5.1 in presence of Bis 4,3. **B,** The temperature dependent (25, 35, 45, 55, and 65°C) CD spectra of 5.1 in presence of TMPyP4. **C,** The temperature dependent (25, 35, 45, 55, and 65°C) CD spectra of 8.1 in presence of Bis 4,3. **D,** The temperature dependent (25, 35, 45, 55, and 65°C) CD spectra of 8.1 in presence of TMPyP4. **E,** The temperature dependent (25, 35, 45, 55, and 65°C) CD spectra of 14.1 in presence of Bis 4,3. **F,** The temperature dependent (25, 35, 45, 55, and 65°C) CD spectra of 14.1 in presence of TMPyP4.

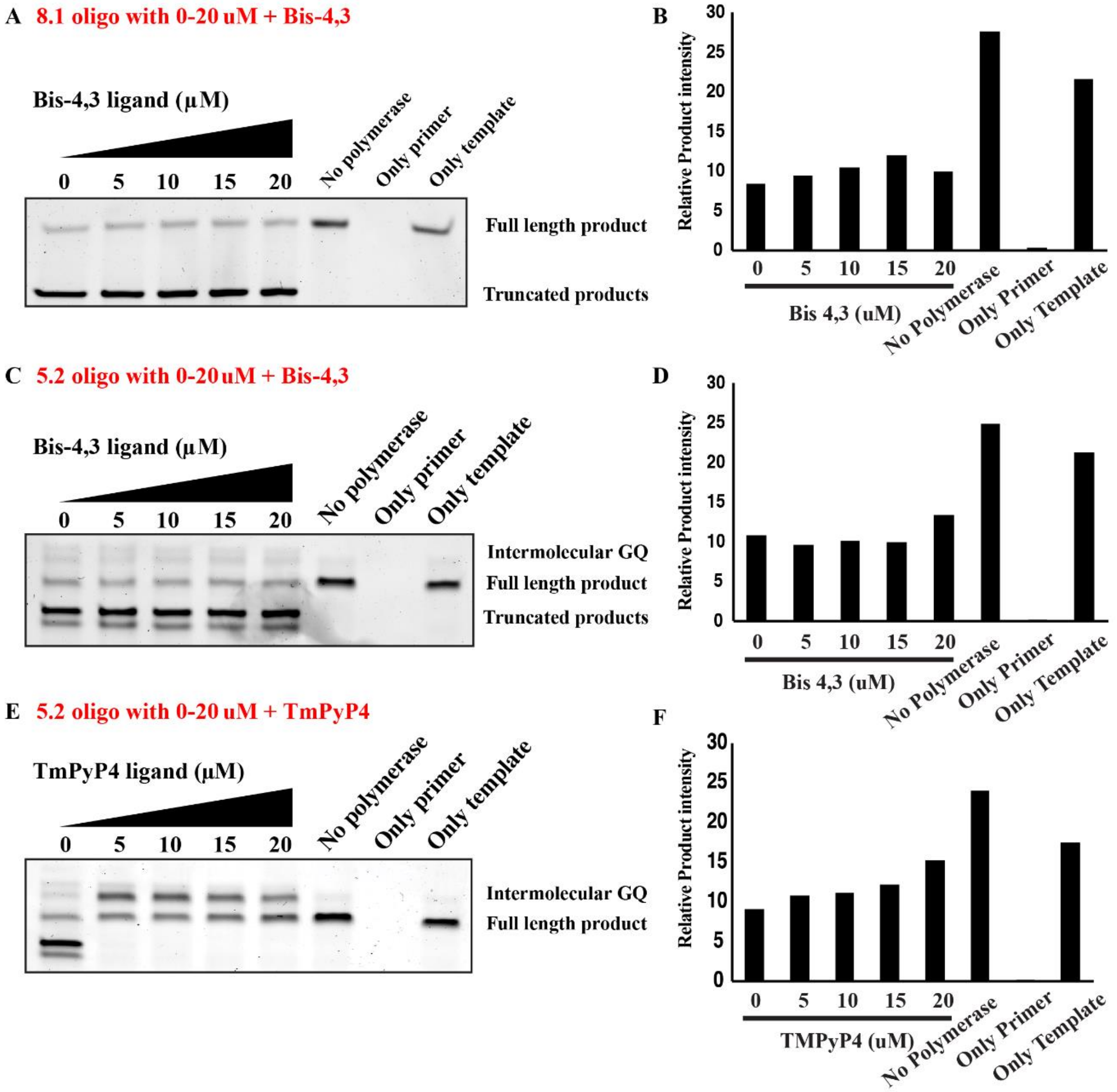

Figure S3. A, showed the produced full-length oligo after primer extension assay of 8.1 in presence of increasing amount (0, 5, 10, 15, and 20 µM) of Bis 4,3 *in vitro*. **B,** the corresponding graphical presentation of amount of full-length product of 8.1 in presence of varying concentration of Bis 4,3. **C,** showed the produced full-length oligo after primer extension assay of 5.2 in presence of increasing amount (0, 5, 10, 15, and 20 µM) of Bis 4,3 *in vitro*. **D,** the graphical presentation of amount of full-length product of 5.2 in presence of varying concentration of Bis 4,3. **C,** showed the produced full-length oligo after primer extension assay of 5.2 in presence of increasing amount (0, 5, 10, 15, and 20 µM) of TMPyP4 *in vitro*. **D,** the graphical presentation of amount of full-length product of 5.2 in presence of varying concentration of TMPyP4.

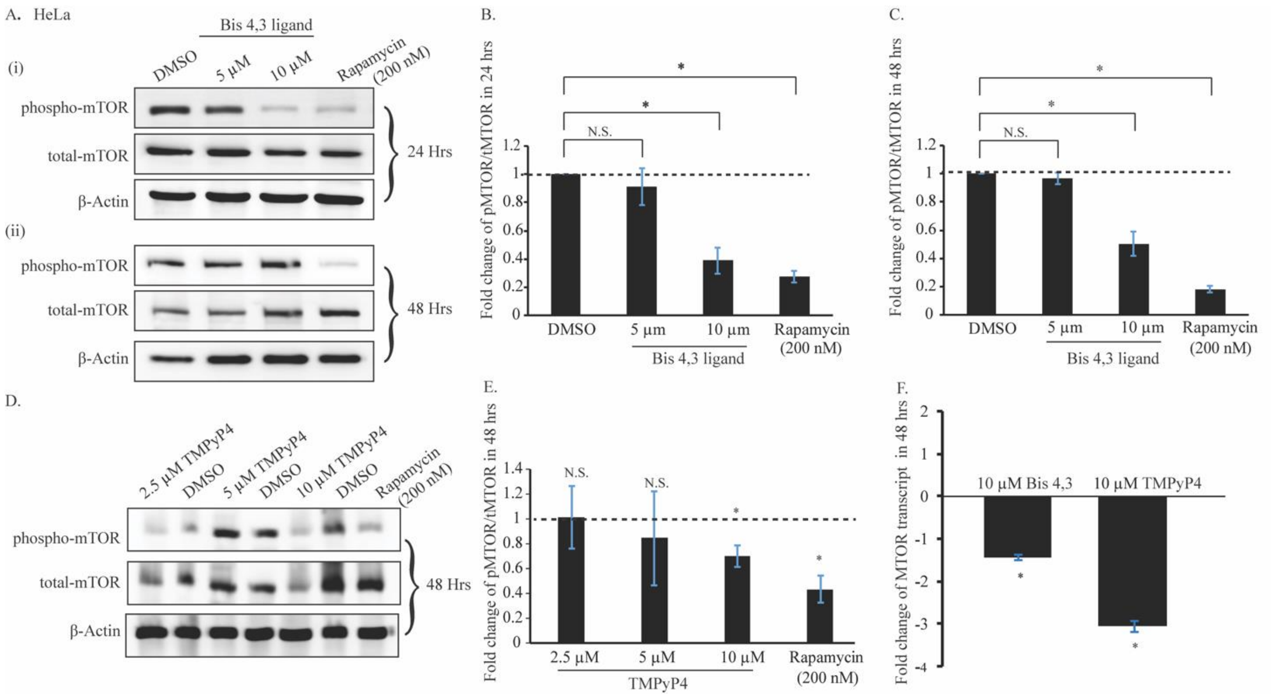

Figure S4: **A,** Western Blot of phospho-mTOR and total mTOR levels in HeLa cells after Bis-4,3 (5 and 10 µM) treatment for **(i)** 24 hours and **(ii)** 48 hours. Rapamycin treatment was used as a positive control. **B,** graphical analysis of the phospho/total mTOR levels of HeLa cells after Bis-4,3 treatment of 24 hours. **C,** graphical analysis of the phospho/total mTOR levels of HeLa cells after Bis-4,3 treatment of 48 hours. **D,** Western Blot of phospho-mTOR and total mTOR level in HeLa cells after TMPyP4 treatment for 48 hours. Rapamycin treatment was used as a positive control. **E,** graphical analysis of the phospho/total mTOR levels of HeLa cells after TMPyP4 treatment for 48 hours. **F,** graphical representation of qRT-PCR ΔΔCT values of mTOR fold change for both Bis-4,3 and TMPyP4 treated condition in HeLa cells for 48 hours compared to respective DMSO treated condition.

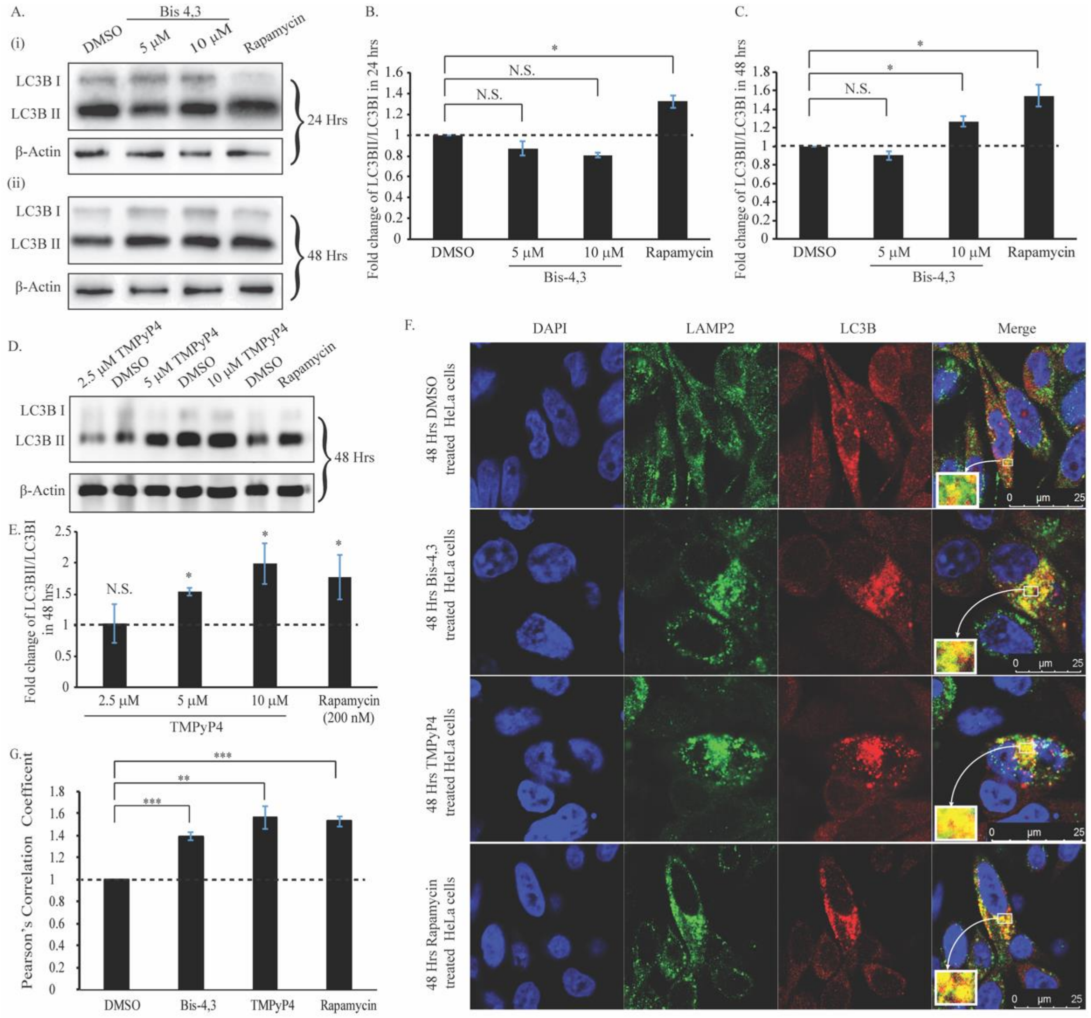

Figure S5: A, Western Blot of LC3B level in HeLa cells after Bis-4,3 treatment for **(i)** 24 hours and **(ii)** 48 hours. Rapamycin treatment was observed as a positive control. **B,** graphical analysis of the LC3BII/LC3BI levels of HeLa cells after Bis-4,3 treatment for 24 hours. **C,** graphical analysis of the LC3BII/LC3BI levels of HeLa cells after Bis-4,3 treatment for 48 hours. **D,** Western Blot of LC3B level in HeLa cells after TMPyP4 treatment for 48 hours. Rapamycin treatment was observed as a positive control. **E,** graphical analysis of the LC3BII/LC3BI levels of HeLa cells after TMPyP4 treatment for 48 hours. **F,** Confocal images of DMSO, Bis-4,3, TMPyP4 and rapamycin-treated HeLa cells. LAMP2 and LC3B proteins are indicated in green and red, respectively. The nucleus was stained with DAPI. **G,** graphical representation of Pearson's correlation coefficient for the co-localization.

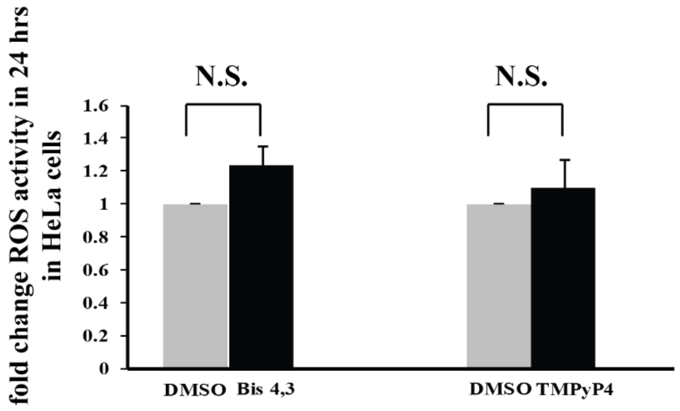

Figure S6: Intracellular ROS activity: The ROS activity was measured in presence of the 10 μM concentration of both Bis 4,3 and TMPyP4 after 48-hours of treatment. The result showed no significant alteration compared to respective DMSO control.

Oligo 8.1 with Bis-4,3

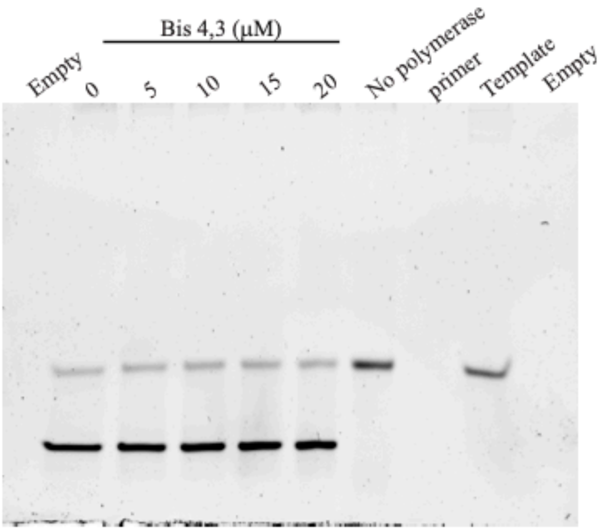

Oligo 5.2 with Bis-4,3

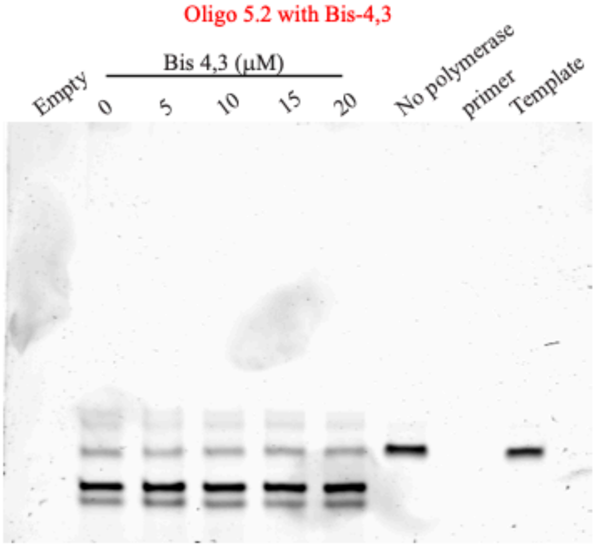

Oligo 5.2 with TMPyP4

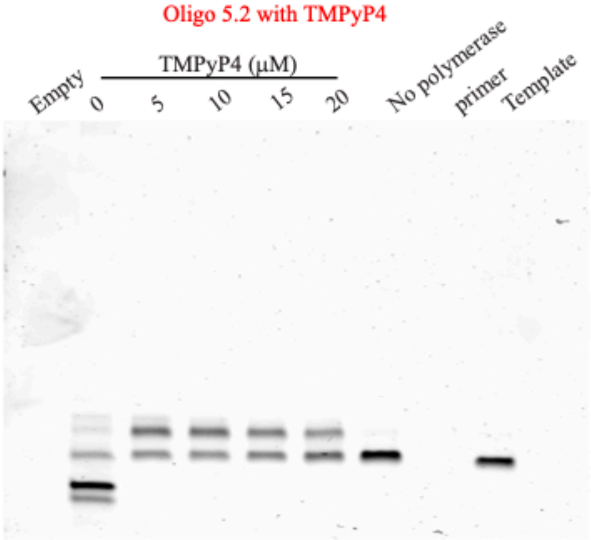

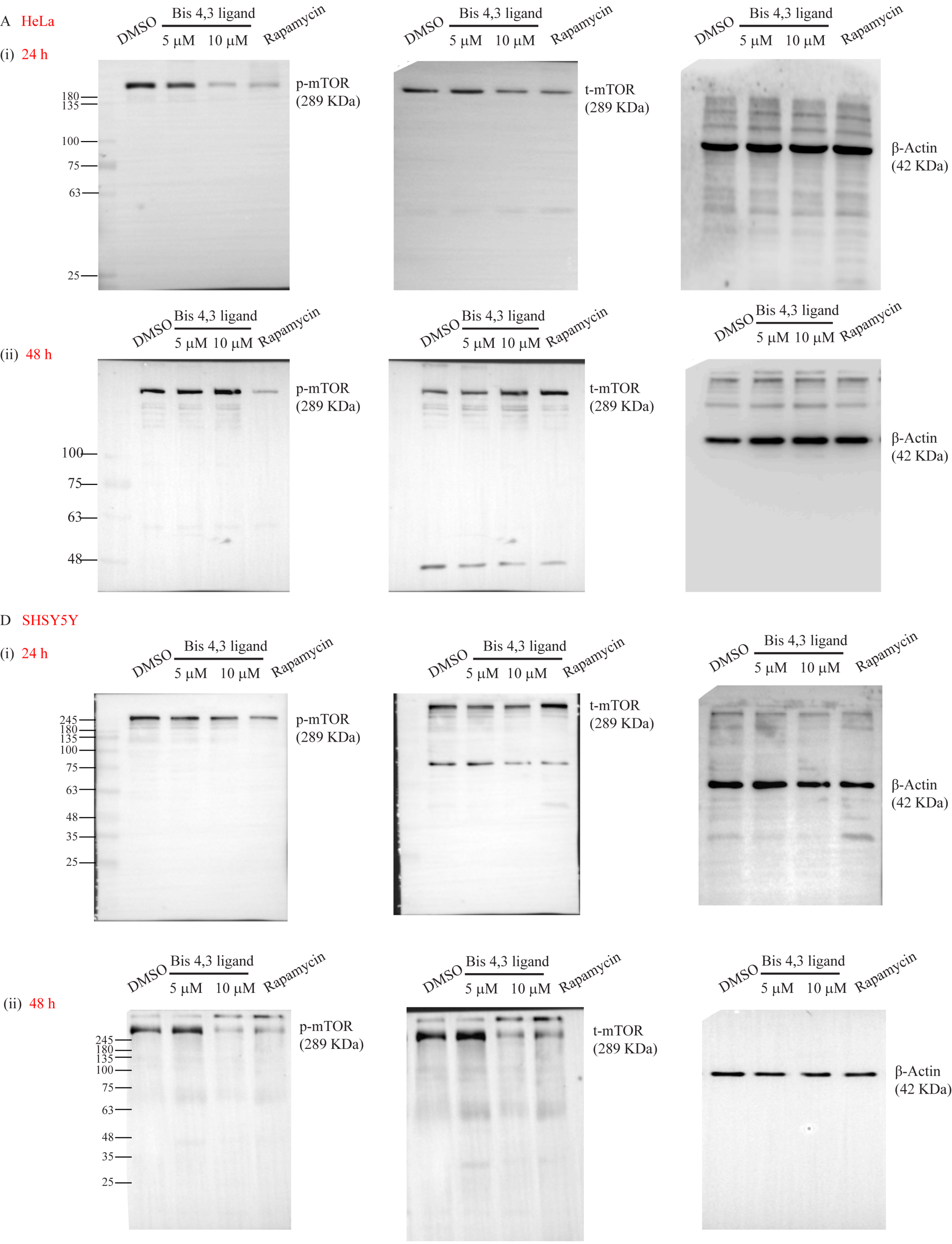

A HeLa
(i) 24 h
DMSO
Bis 4,3 ligand
5 µM
10 µM
Rapamycin
p-mTOR
(289 KDa)
t-mTOR
(289 KDa)
β-Actin
(42 KDa)
180
135
100
75
63
25
(ii) 48 h
100
75
63
48
D SHSY5Y
(i) 24 h
245
180
135
100
75
63
48
35
25
(ii) 48 h

G HeLa

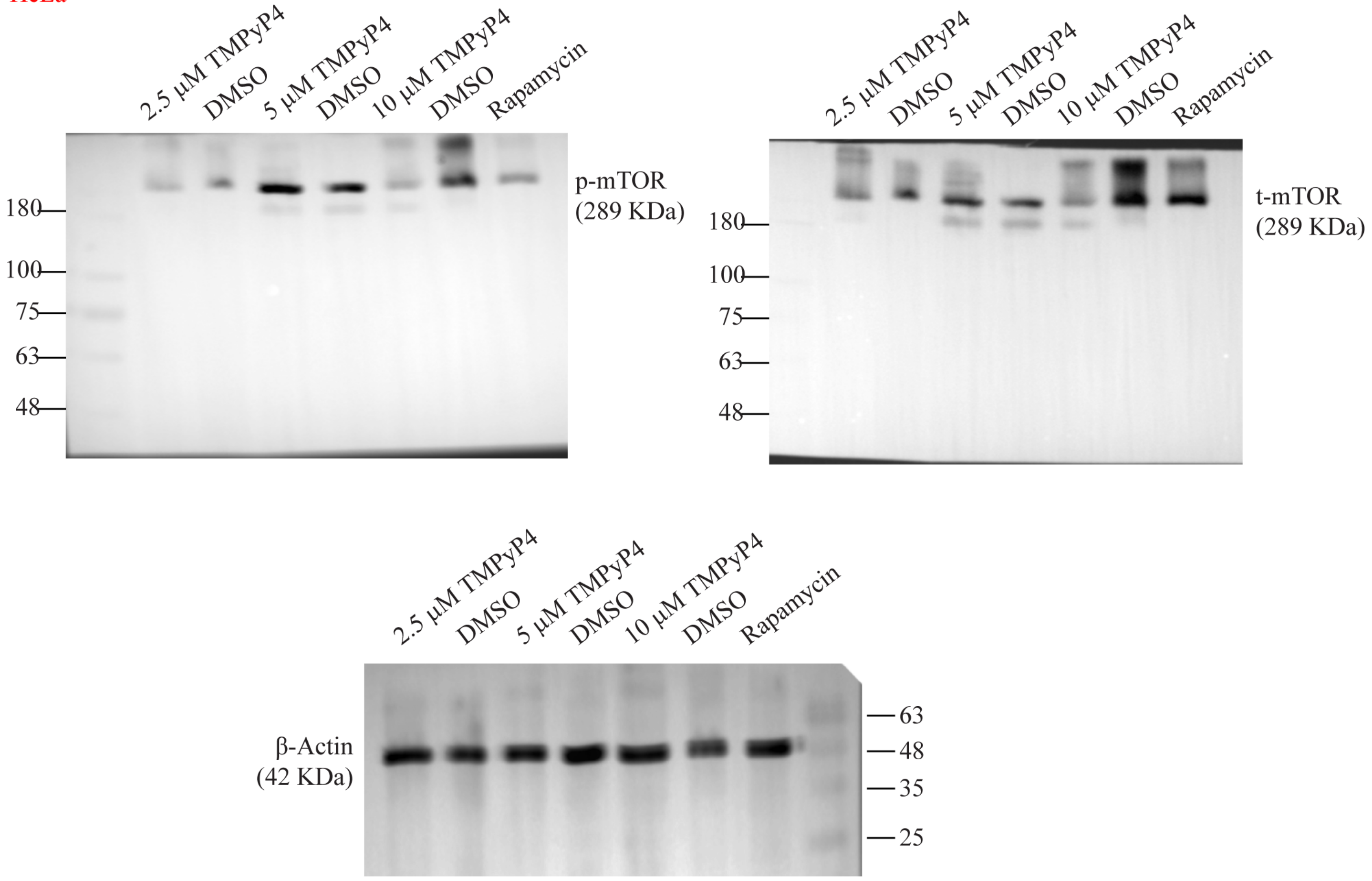

H SHSY5Y

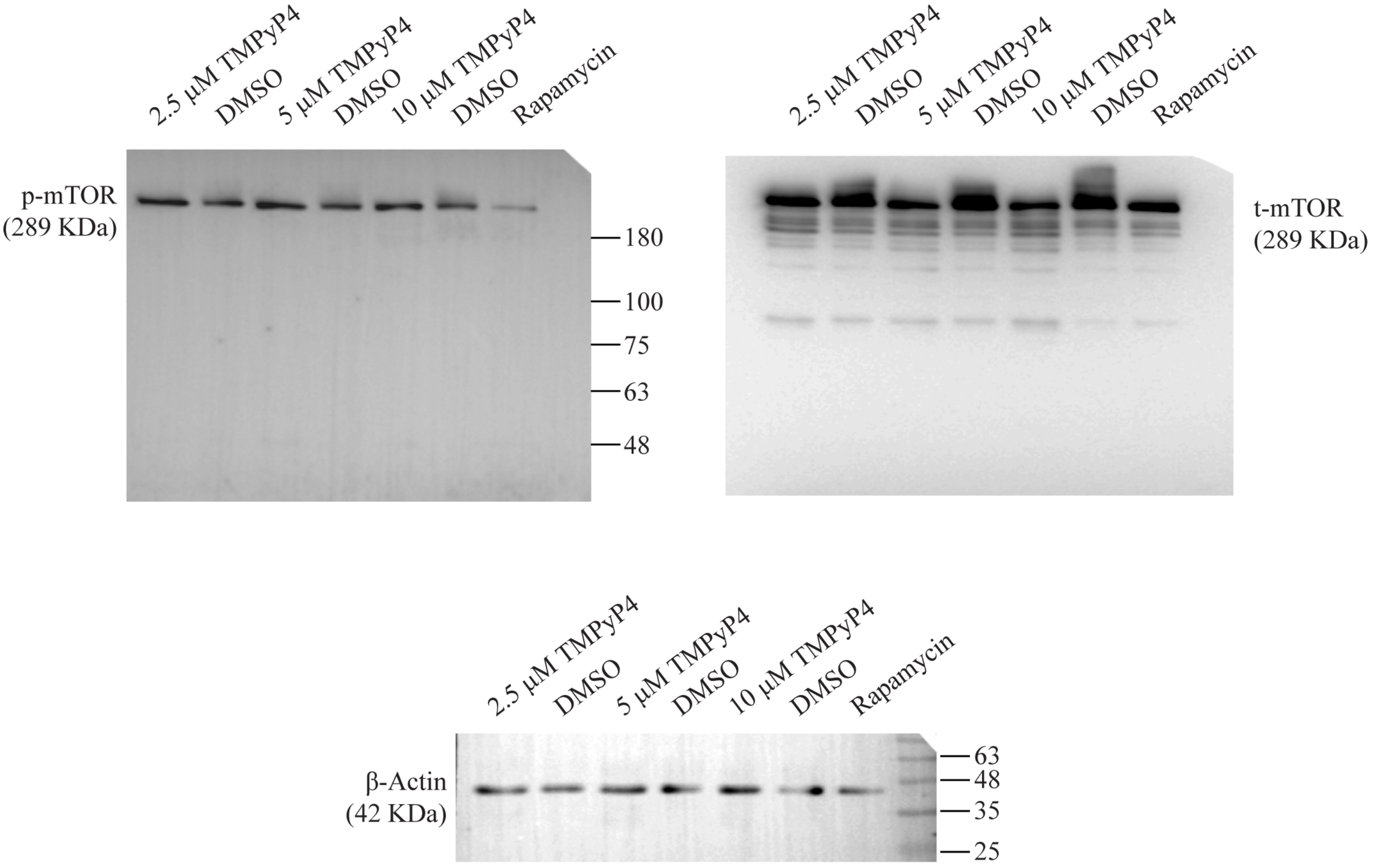

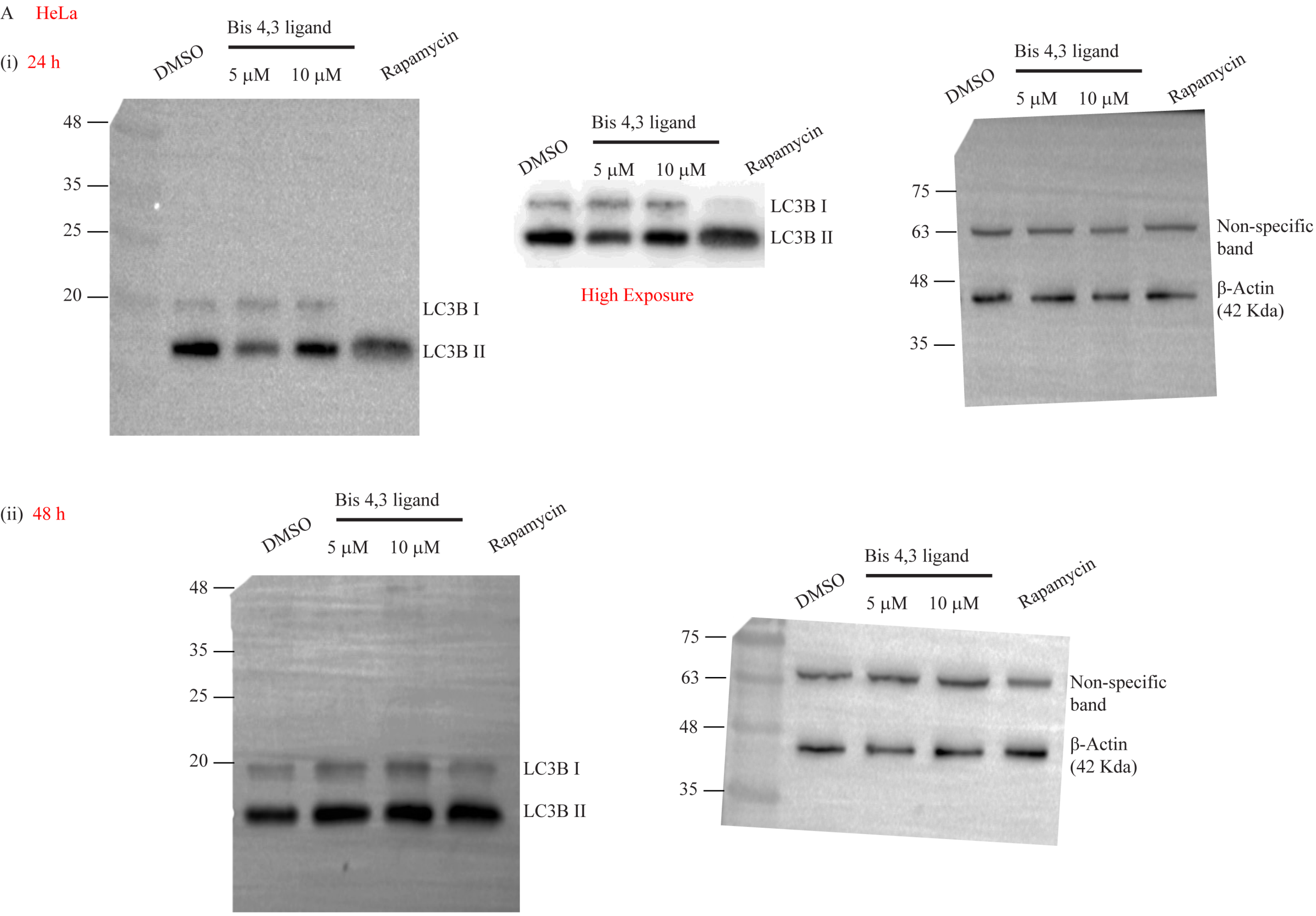
A HeLa
(i) 24 h
Bis 4,3 ligand
DMSO
5 µM
10 µM
Rapamycin
48
35
25
20
LC3B I
LC3B II
High Exposure
75
63
Non-specific band
β-Actin (42 Kda)
(ii) 48 h

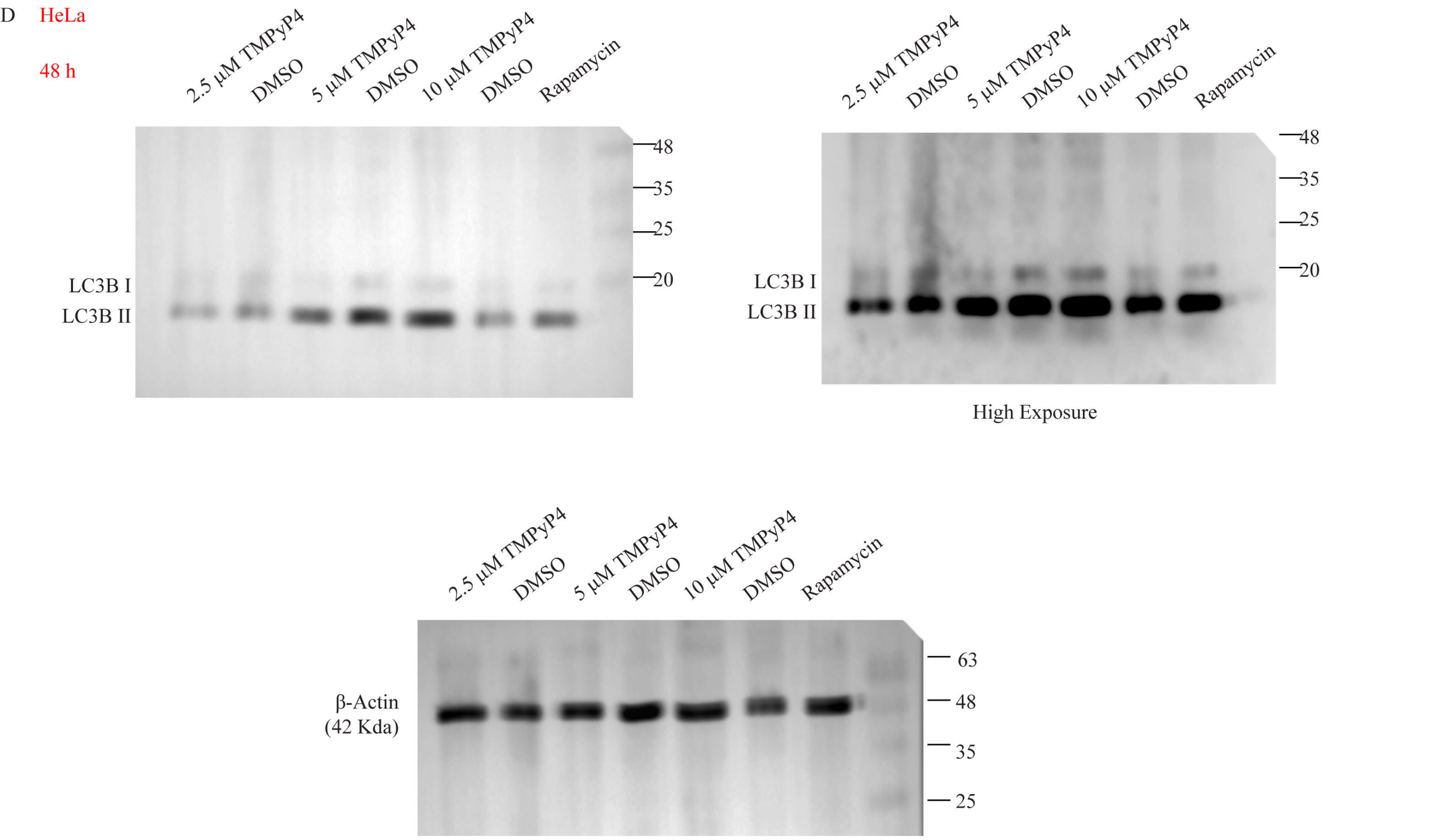
D HeLa
48 h
2.5 µM TMPyP4
DMSO
5 µM TMPyP4
DMSO
10 µM TMPyP4
DMSO
Rapamycin
LC3B I
LC3B II
48
35
25
20
High Exposure
β-Actin (42 Kda)
63

A SHSY5Y

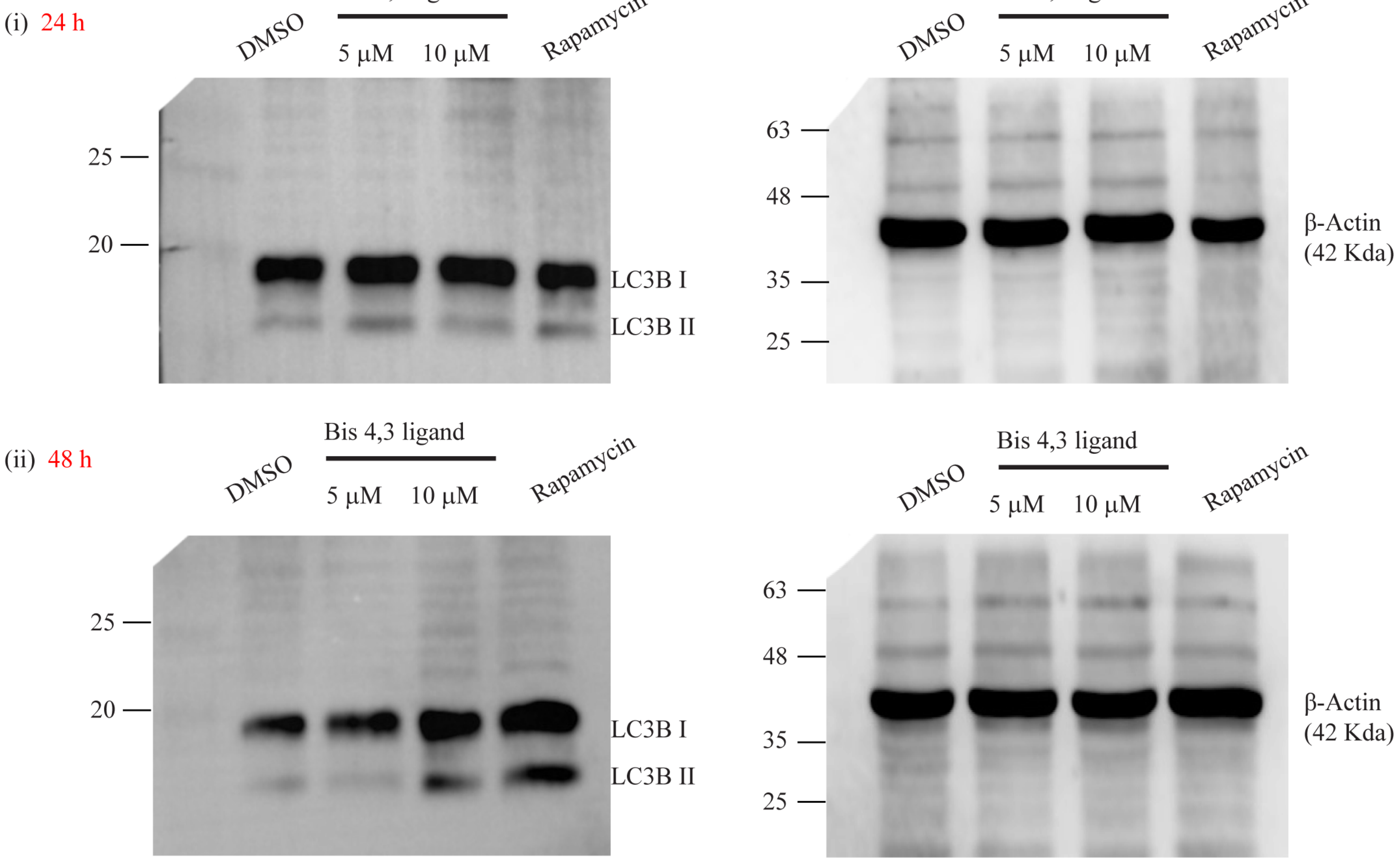

D SHSY5Y

48 h

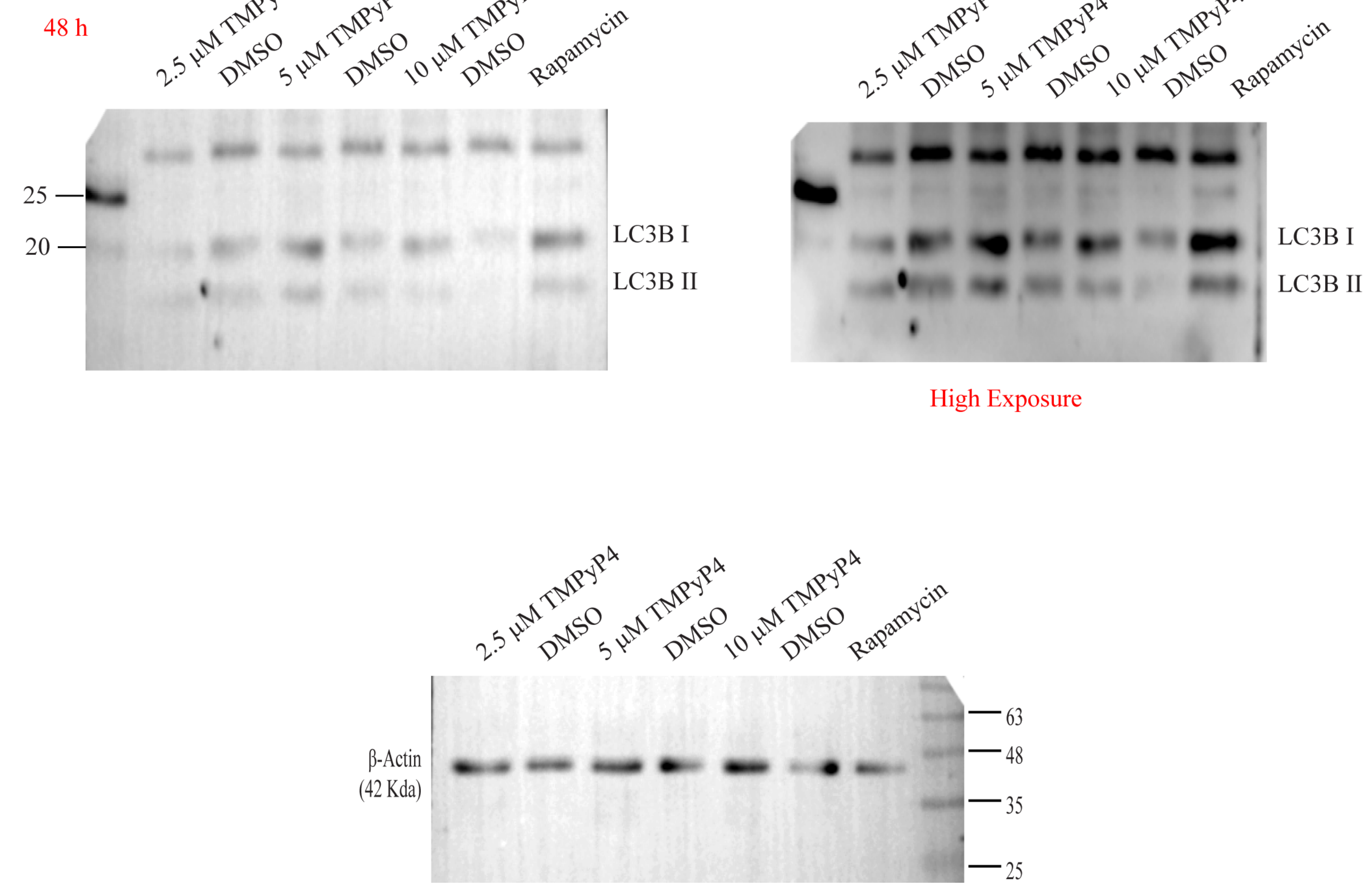

Bis 4,3 treatment on Hela cells for 24 hours

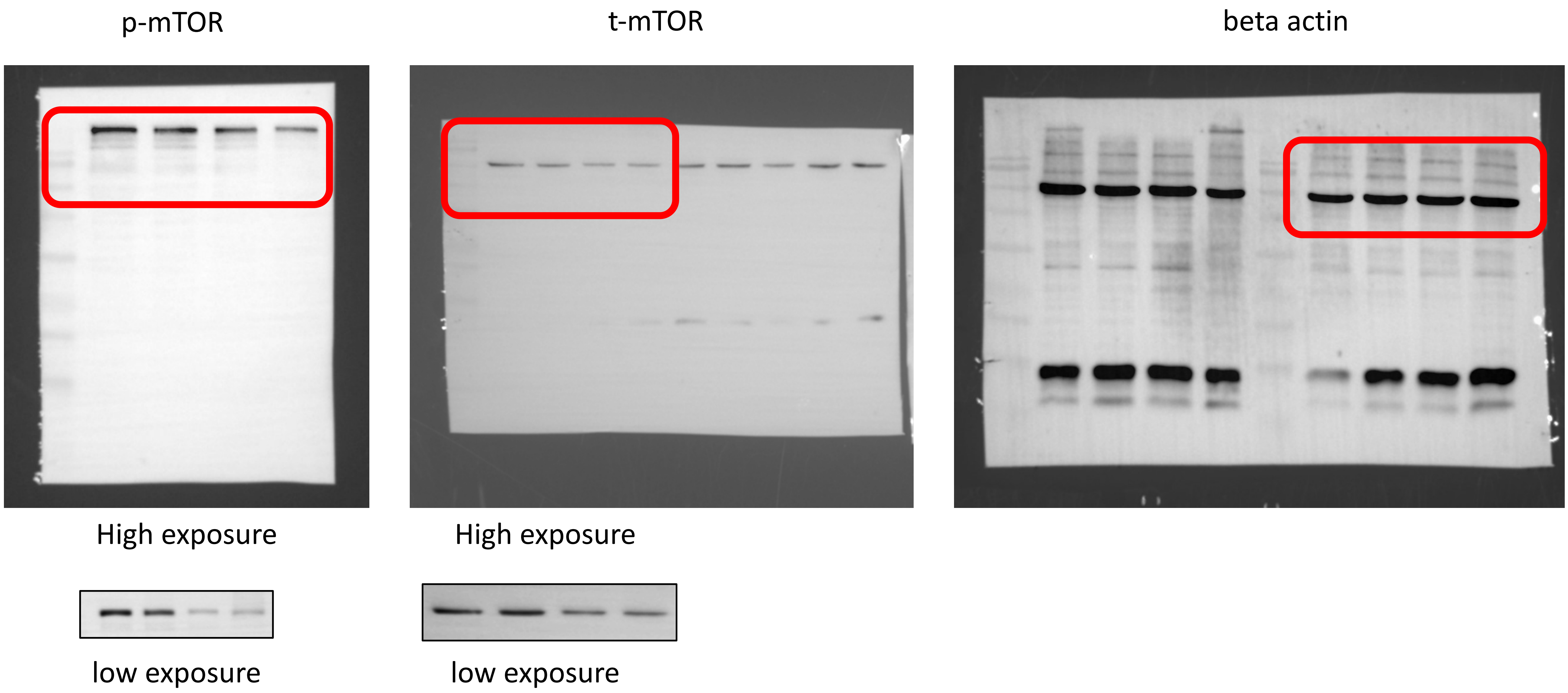

Bis 4,3 treatment on Hela cells for 48 hours

p-mTOR

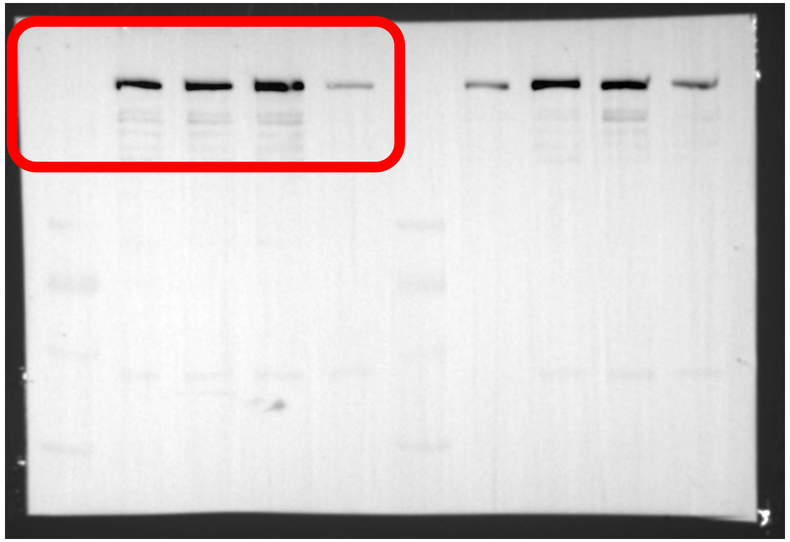

t-mTOR

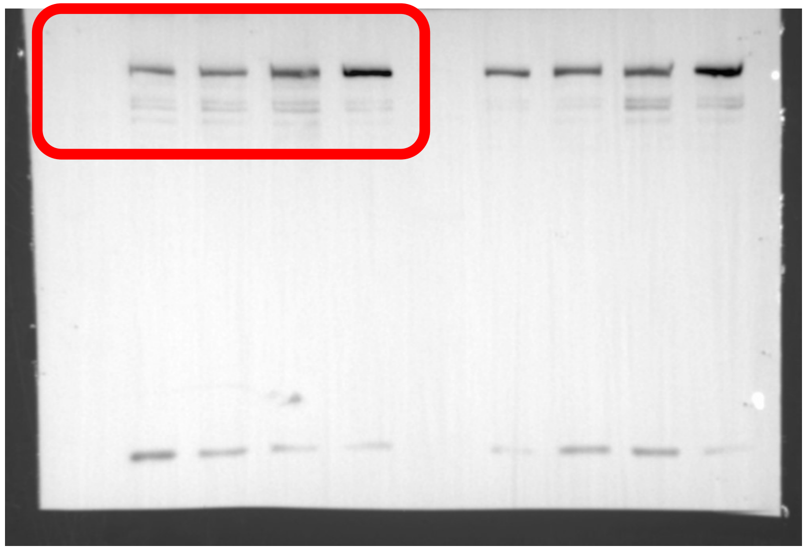

beta actin

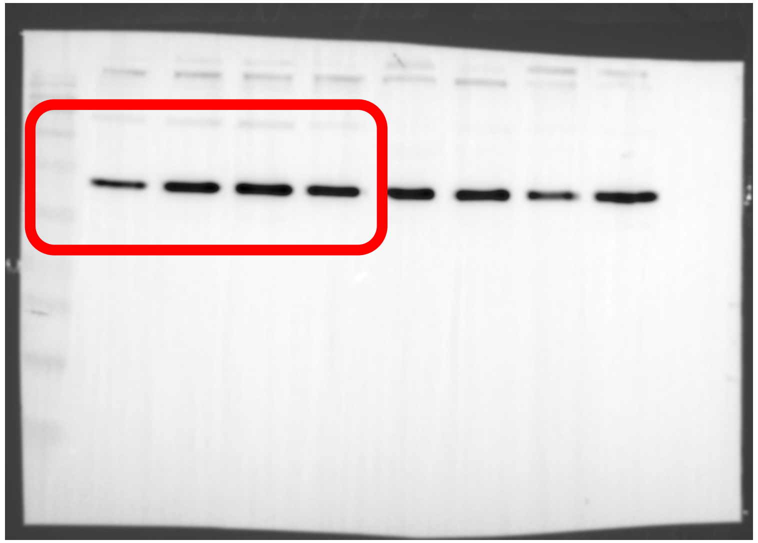

Bis 4,3 treatment on SHSY55Y cells for 24 hours

p-mTOR

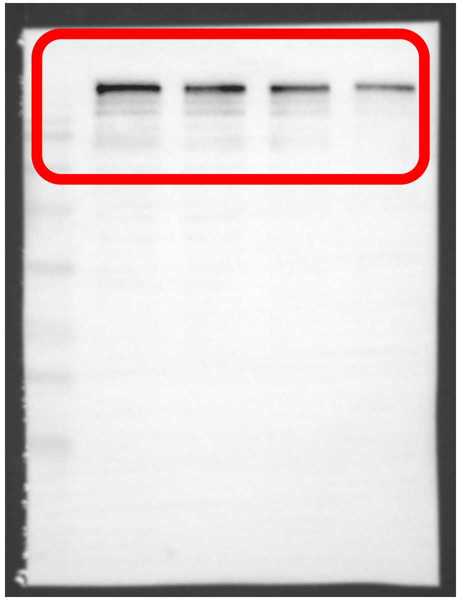

t-mTOR

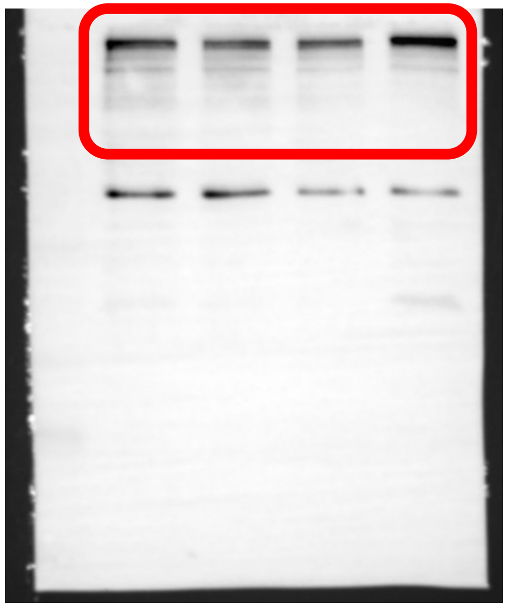

beta actin

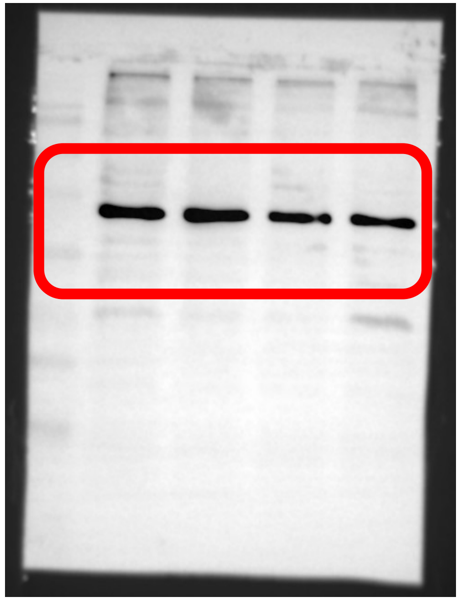

Bis 4,3 treatment on SHSY55Y cells for 48 hours

p-mTOR

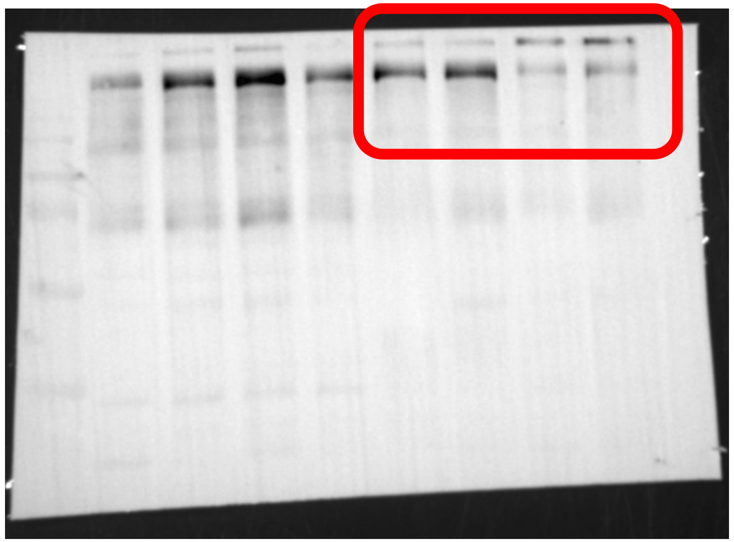

t-mTOR

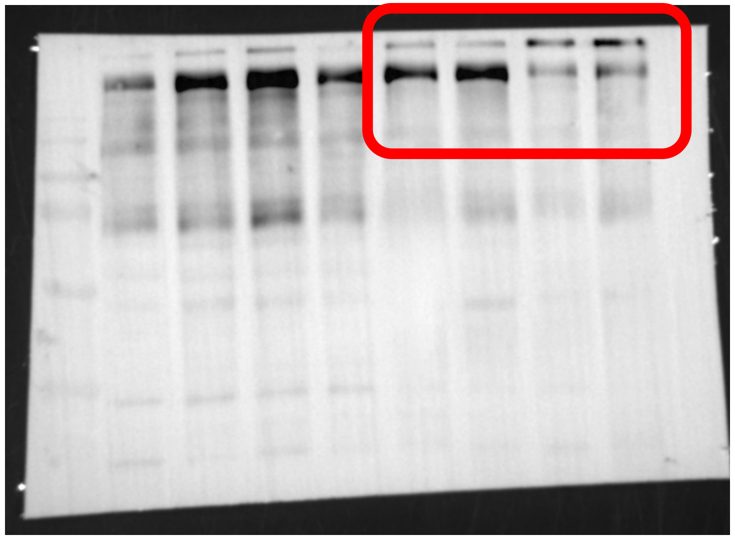

beta-actin

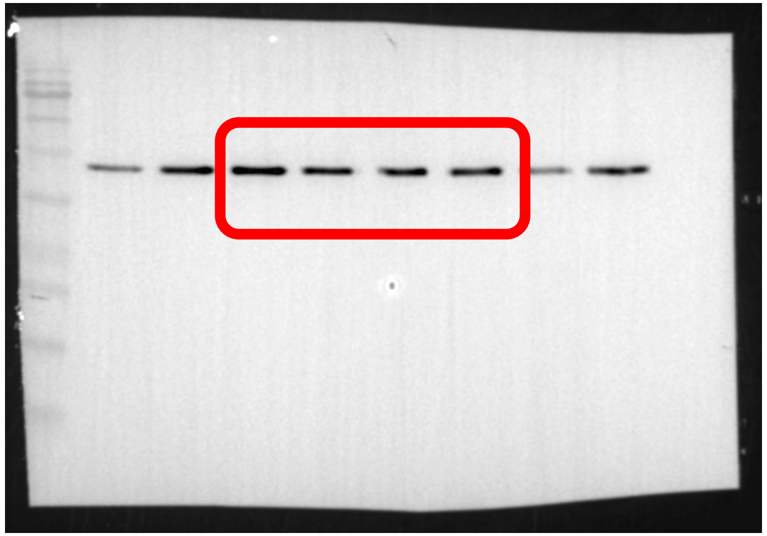

TMPyP4 treatment on Hela cells for 48 hours

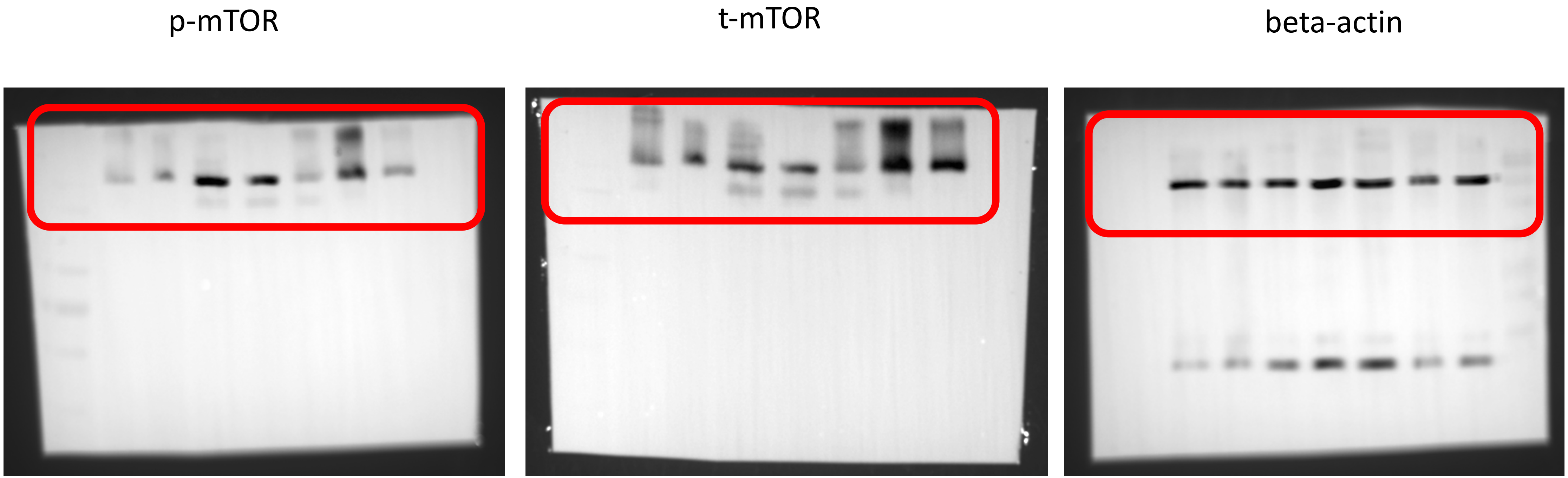

TMPyP4 treatment on SHSY5Y cells for 48 hours

p-mTOR

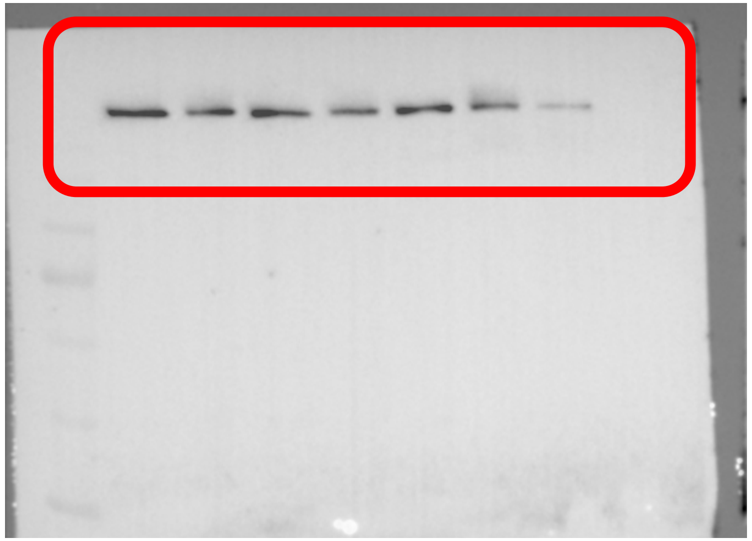

low exposure

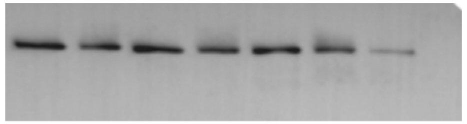

high exposure

t-mTOR

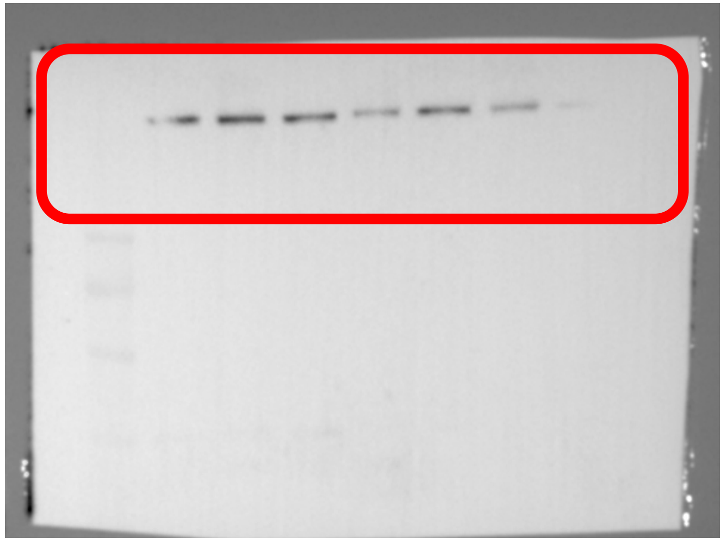

low exposure

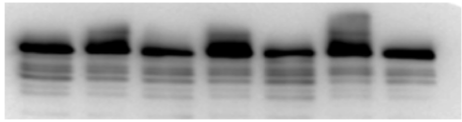

high exposure

beta-actin

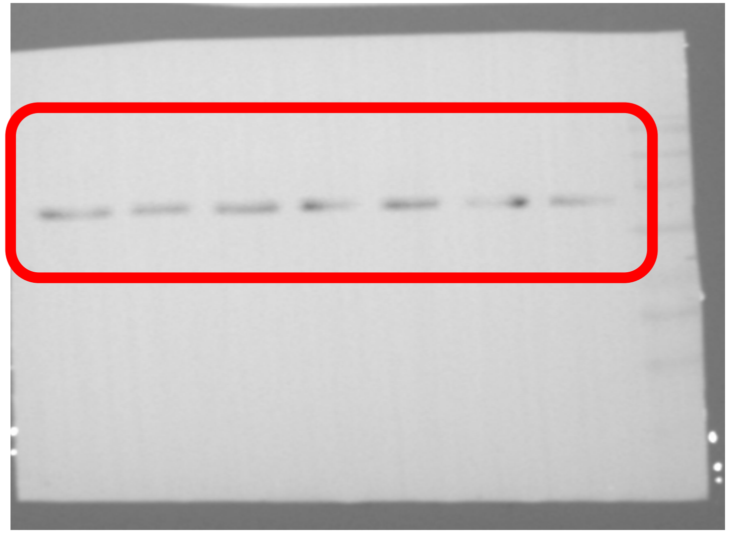

Bis 4,3 treatment on Hela cells for 24 hours

LC3BI and LC3BII

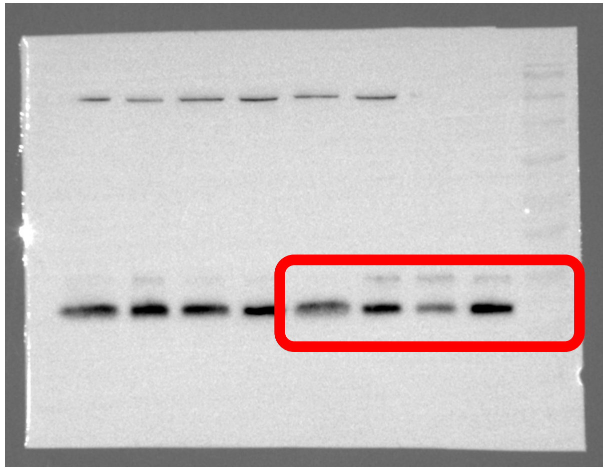

beta-actin

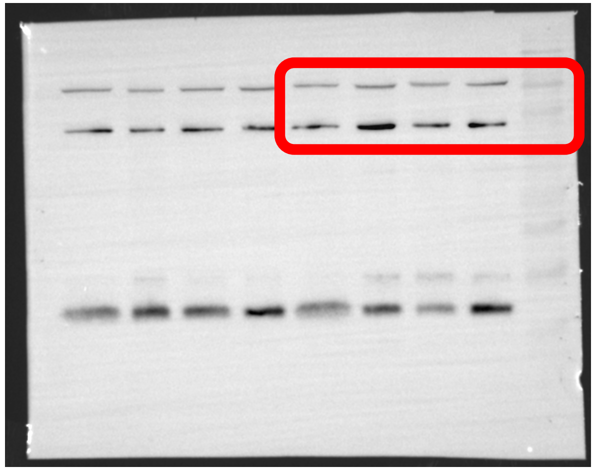

Bis 4,3 treatment on Hela cells for 48 hours

LC3BI and LC3BII

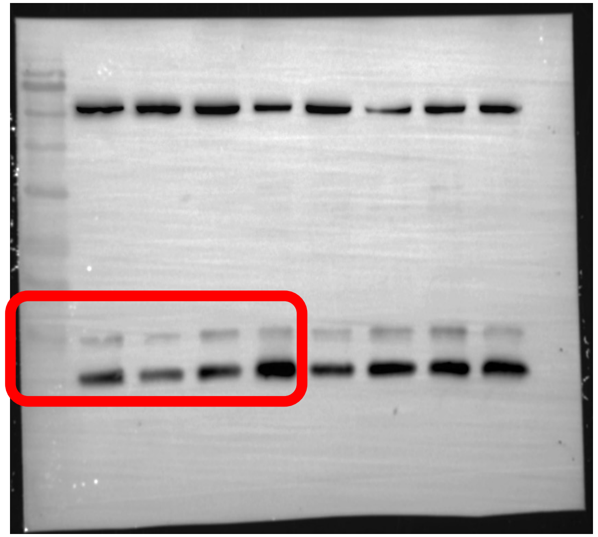

beta-actin

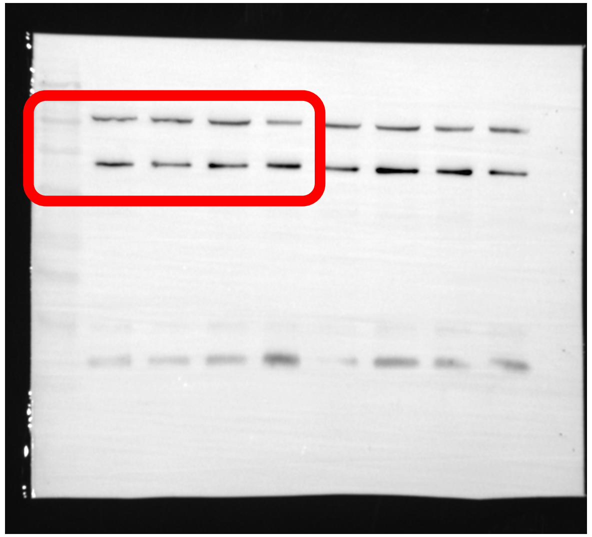

TMPyP4 treatment on Hela cells for 48 hours

LC3BI and LC3BII

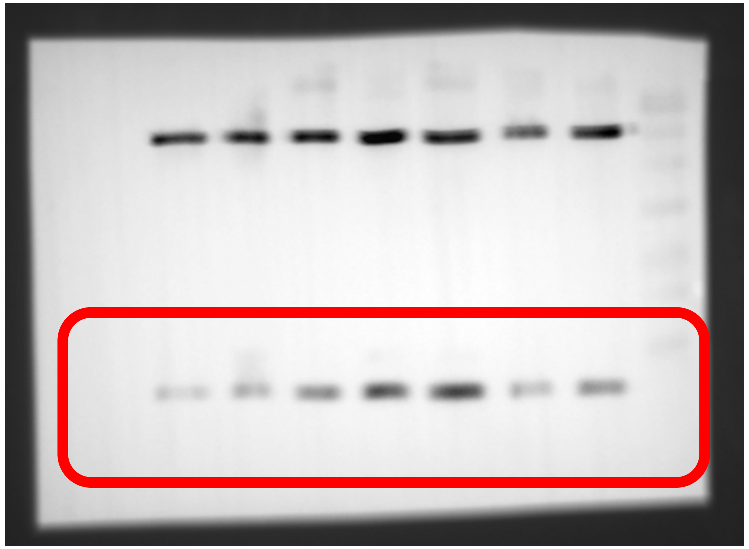

beta-actin

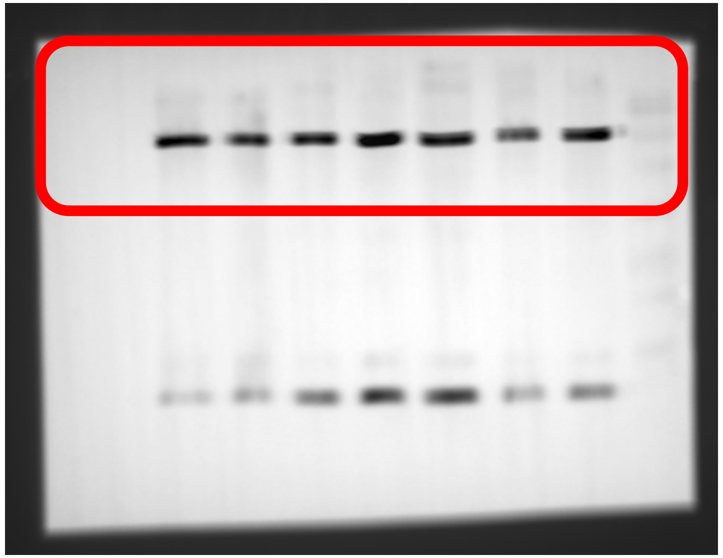

Bis 4,3 treatment on SHSY55Y cells for 24 hours

LC3BI and LC3BII

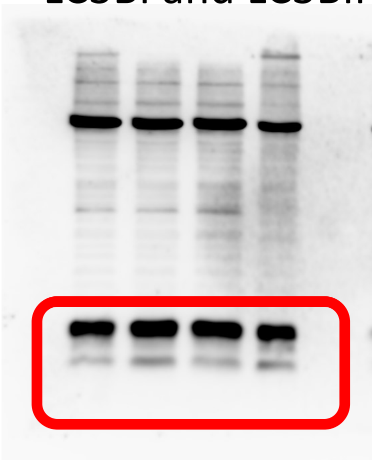

beta-actin

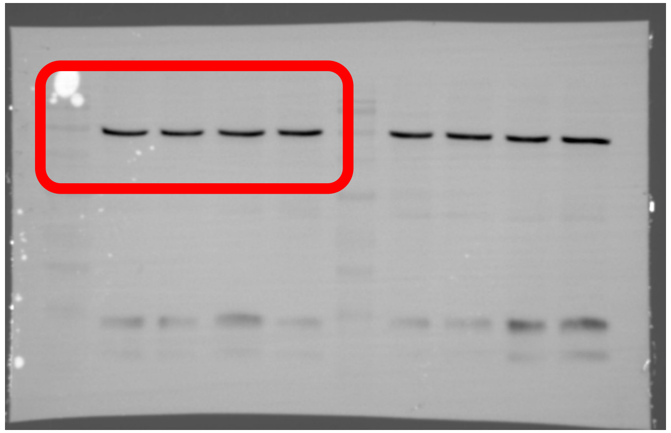

Bis 4,3 treatment on SHSY55Y cells for 48 hours

LC3BI and LC3BII

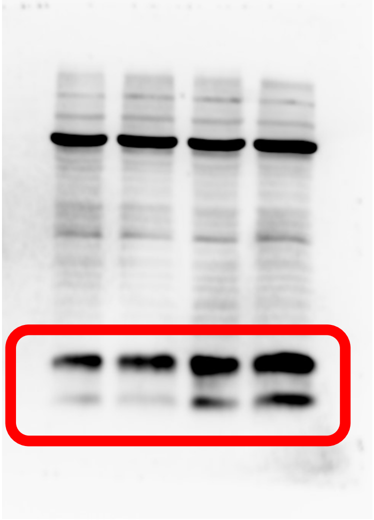

beta-actin

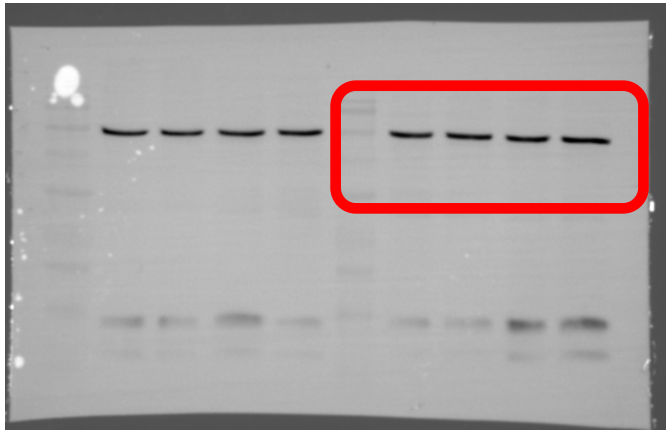

TMPyP4 treatment on SHSY5Y cells for 48 hours

LC3BI and LC3BII

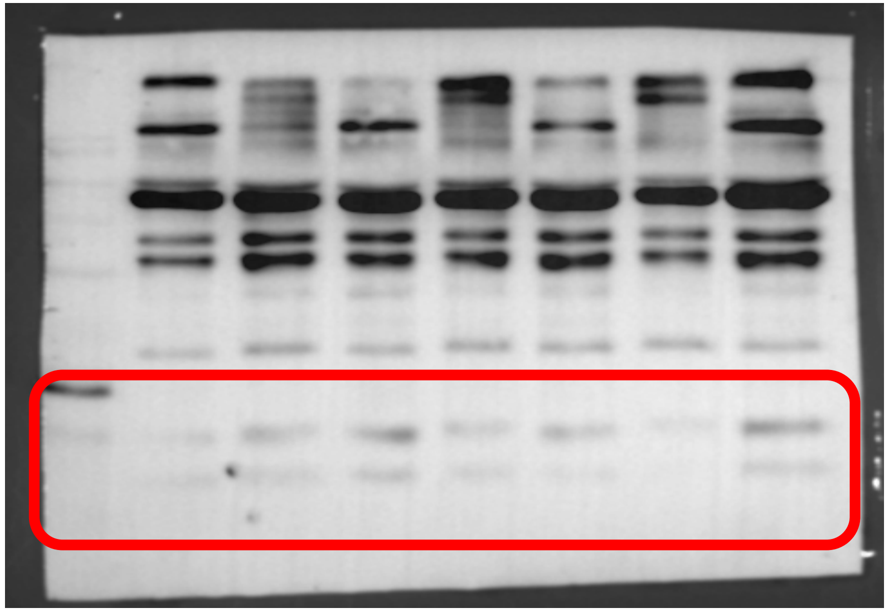

beta-actin

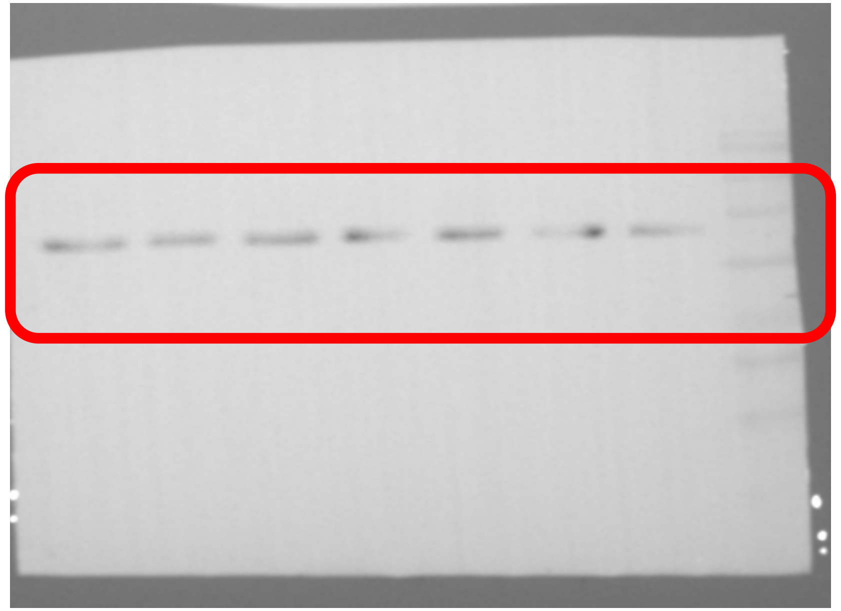

Supplement: Supplementary file 1 — Supplementary Information. [file 41598_2024_52561_MOESM1_ESM.pdf]
